# Identification and characterization of a nonbiological small-molecular mimic of a Zika virus conformational neutralizing epitope

**DOI:** 10.1073/pnas.2312755121

**Published:** 2024-05-14

**Authors:** Priscila M. S. Castanha, Patrick J. McEnaney, Yongseok Park, Anthea Bouwer, Elton J. F. Chaves, Roberto D. Lins, Nicholas G. Paciaroni, Paige Dickson, Graham Carlson, Marli T. Cordeiro, Tereza Magalhaes, Jodi Craigo, Ernesto T. A. Marques, Thomas Kodadek, Donald S. Burke

**Affiliations:** ^a^Department of Infectious Diseases and Microbiology, School of Public Health, University of Pittsburgh, Pittsburgh, PA 15261; ^b^Department of Chemistry, The Herbert Wertheim University of Florida Scripps Institute for Biomedical Innovation and Technology, Jupiter, FL 33458; ^c^Department of Biostatistics, School of Public Health, University of Pittsburgh, Pittsburgh, PA 15261; ^d^Department of Microbiology and Molecular Genetics, School of Medicine, University of Pittsburgh, Pittsburgh, PA 15219; ^e^Department of Virology, Aggeu Magalhaes Institute, Oswaldo Cruz Foundation, Cidade Universitearia, Recife, Pernambuco 50740-465, Brazil; ^f^Deluge Biotechnologies, Jupiter, FL 33458; ^g^Department of Entomology, Texas A&M University, College Station, TX 77843; ^h^Department of Preventive and Social Medicine, School of Medicine, Universidade Federal da Bahia, Bahia 40026-010, Brazil; ^i^Department of Epidemiology, School of Public Health, University of Pittsburgh, Pittsburgh, PA 15261

**Keywords:** Zika, diagnostic, epitope surrogate, flavivirus, non-natural oligomers

## Abstract

Antigenic similarities between Zika virus (ZIKV) and other flaviviruses pose challenges to the development of virus-specific diagnostic tools and effective vaccines. Starting with a DNA-encoded one-bead-one-compound combinatorial library of 508,032 synthetic, non-natural oligomers, we selected and characterized small molecules that mimic ZIKV epitopes. High-throughput fluorescence-activated cell sorter-based bead screening was used to select molecules that bound IgG from ZIKV-immune but not from dengue-immune sera. Deep sequencing of the DNA from the “Zika-only” beads identified 40 candidate molecular structures. A lead candidate small molecule “CZV1-1” was selected that correctly identifies serum specimens from Zika-experienced patients with good sensitivity and specificity (85.3% and 98.4%, respectively). Binding competition studies of purified anti-CZV1-1 IgG against known ZIKV-specific monoclonal antibodies (mAbs) showed that CZV1-1 mimics a nonlinear, neutralizing conformational epitope in the domain III of the ZIKV envelope. Purified anti-CZV1-1 IgG neutralized infection of ZIKV in cell cultures with potencies comparable to highly specific ZIKV-neutralizing mAbs. This study demonstrates an innovative approach for identification of synthetic non-natural molecular mimics of conformational virus epitopes. Such molecular mimics may have value in the development of accurate diagnostic assays for Zika, as well as for other viruses.

## Significance

Zika virus (ZIKV) remains a global health threat of high priority. ZIKV is closely related to the dengue viruses, and its spread in dengue-endemic areas poses significant challenges to the development of virus-specific diagnostic tools and effective vaccines. We screened combinatorial libraries of small synthetic molecules to efficiently identify a nonbiological molecule “CZV1-1” that binds specifically to IgG present in serum from Zika-immune persons, but not in serum from dengue-immune persons. CZV1-1 mimics a major Zika-neutralizing envelope epitope and can serve as a biomarker for evidence of prior Zika infection in flavivirus-endemic areas. This approach can be used to find small-molecule mimics of important epitopes for a wide range of other infectious and noninfectious diseases.

Zika virus (ZIKV) is a global health threat that causes adverse pregnancy and fetal outcomes, as well as Guillain–Barre syndrome in healthy adults (1). Reduced but nonetheless sustained, the transmission of ZIKV has been documented in recent years, particularly in regions that are highly endemic for other mosquito-borne flaviviruses such as dengue viruses (DENV1-4) (2–4). Antigenic similarities between ZIKV and DENV lead to the development of both virus-specific and cross-reactive antibodies following infection with either of these viruses (5). Immune interactions can modulate transmission and risk for subsequent enhanced disease by ZIKV and DENV (6–8), which poses challenges to the development of virus-specific diagnostic tools and effective vaccines.

Recently, there has been considerable interest in the identification of unique antigenic determinants for highly specific detection and differentiation of flavivirus infections as well as for defining immune correlates of protection against ZIKV (9–12). We report here an innovative approach to this problem using “epitope surrogate” technology, which allows the unbiased identification of IgG antibodies that distinguish two patient populations. This technique has been employed previously to identify biomarker antibodies unique to patients with an active (versus a latent) tuberculosis infection, antibodies resulting from an autoimmune condition (Type 1 diabetes), among other applications (13–15).

The method involves labeling IgGs in serum samples collected from case and control patient populations with different colored fluorescent dyes by preincubation with dye-conjugated single-chain anti-IgG antibodies. The serum samples are then mixed and incubated with a one-bead-one-compound (OBOC) DNA-encoded library (DEL) of synthetic, nonpeptidic molecules. These libraries are synthesized by combinatorial chemistry and DNA tagged on microbeads. Each library includes oligomers of a wide variety of organic compound precursors that are built randomly to create shapes and diverse chemical functional groups beyond the canonical 20 amino acids (14). After washing, the beads are passed through a fluorescence-activated cell sorter (FACS) gated to collect beads that display a high level of fluorescence in the “case channel” but a low level of fluorescence in the “control channel”. The assumption is that these beads display a ligand for the antigen-binding site of antibodies that are present at much higher levels in the case sera than the control sera. We call these ligands epitope surrogates. In addition to providing an unbiased method for the identification of antibodies that differentiate two patient populations, epitope surrogate technology has the additional advantage of providing a pathway to identify the native antigen recognized by the differentiating antibodies. This involves using an immobilized epitope surrogate as an affinity reagent to enrich from the serum the IgGs to which it binds. These enriched IgGs can then be used to identify the native antigen they recognize by either testing purified candidate antigens in enzyme-linked immunosorbent assay (ELISA) or by carrying out immunoprecipitation from extracts that contain the native antigen, then identifying the retained proteins by mass spectrometry.

Here, we screened an OBOC DEL of peptoid-inspired conformationally constrained oligomers (PICCOs) (16) against differentially labeled serum samples collected from individuals infected with either DENV or ZIKV. We performed next-generation sequencing (NGS) of the encoding tags on beads that retained high levels of antibodies from the “ZIKV-only” patients and low levels of antibodies from the “DENV-only” patients. Clustering analysis of structurally homologous hits that reacted with Zika-only sera identified 40 PICCOs that were candidate epitope surrogates engaging ZIKV-specific antibodies. In this study, we report the detailed characterization of one of these screening hits, which we call “CZV1-1”. FACS-based screening of a larger panel of acute and convalescent serum samples from patients with a repertoire of flavivirus immune reactivity revealed that CZV1-1 classified patients as Zika-immune with a sensitivity and specificity of 85.3% (95% CI: 69.8 to 93.5) and 98.4% (95% CI: 95.5 to 99.6), respectively. Affinity chromatography using immobilized CZV1-1 resulted in a ≈600-fold enrichment of the antibodies from the serum that recognize this synthetic molecule. These enriched IgGs were then tested for binding to various candidate antigens, which revealed they recognize domain III (DIII) of the ZIKV envelope protein. These CZV1-1-binding antibodies show minimal reactivity to DIII of DENV1-4 envelope proteins. Competitive binding assays using CZV1-1 specific IgG and several ZIKV-specific monoclonal antibodies (mAbs) identified previously demonstrated that CZV1-1-binding antibodies recognize an epitope in the ZIKV envelope DIII C-C’ loop region. Finally, CZV1-1 specific IgG neutralized infection of distinct strains of ZIKV (Brazil and French Polynesia) with a per milligram potency comparable to that of highly specific ZIKV mAbs. These results demonstrate that systematic mining of “antigenically agnostic” libraries of nonbiological small molecules (that is, molecular libraries constructed with no reference to known virus sequences, structures, or antigens) can nonetheless be used to identify biomarker correlates of virus neutralization.

## Results

### Identification of Epitope Surrogates that Bind ZIKV-Specific Antibodies.

**Fig. 1.**
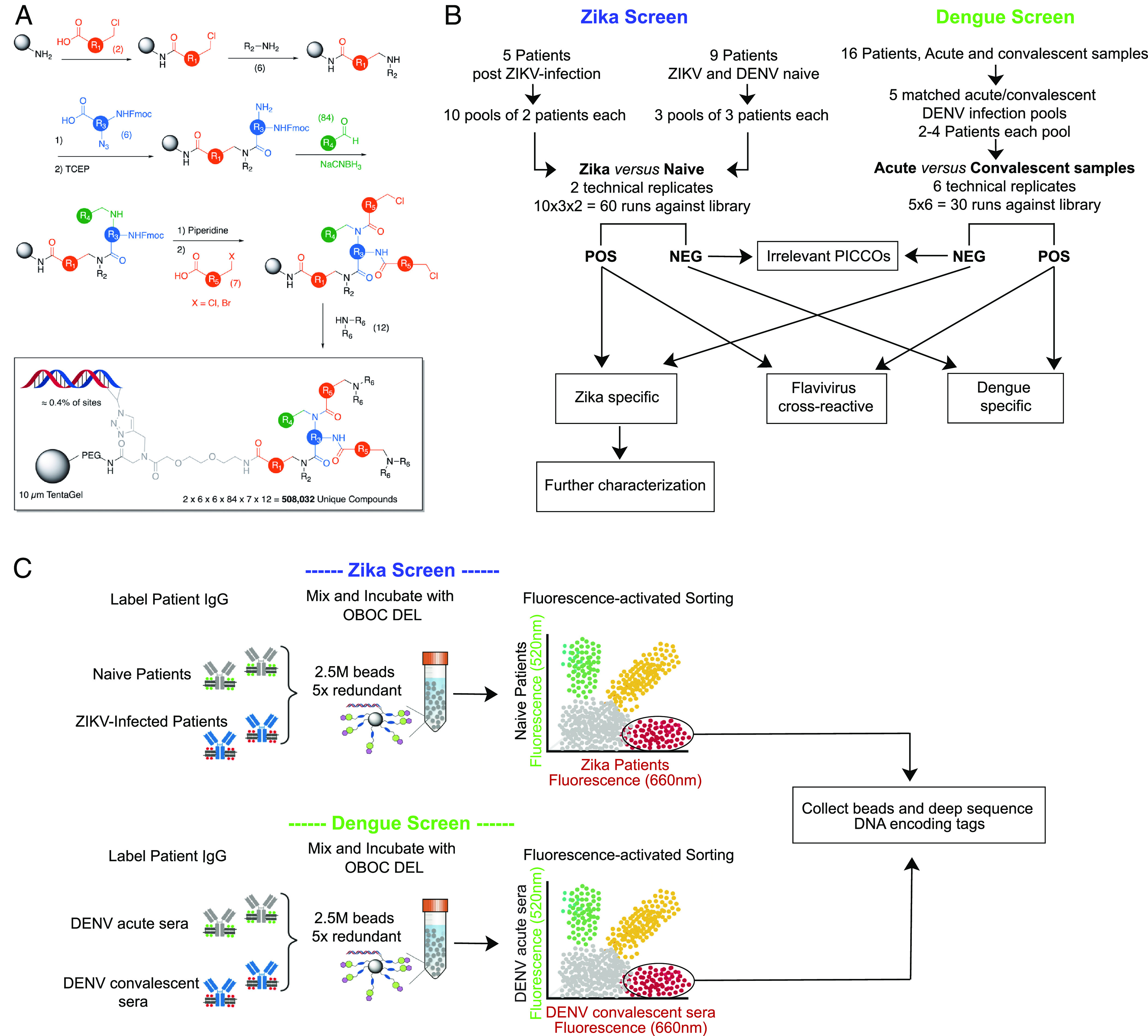
A OBOC DEL to select for small molecules that mimic ZIKV epitopes. (*A*) Schematic representation of the synthesis of the OBOC DEL. Split and pool solid-phase synthesis was employed at each step. 10 µm TentaGel beads (gray oval) were first acylated with one of two chloroacids (red). The chloride was then displaced by one of six primary amines. The resultant secondary amine was acylated with one of six azide-containing, Fmoc-protected amino acids (blue). After reduction of the azide with TCEP, the resultant amine was reductively alkylated with one of 85 aldehydes and NaCNBH3. The Fmoc group was removed, and the two amines were then acylated with one of 7 haloacids (red). Finally, the halide was displaced by a secondary amine to provide the final DEL. In the box, the DNA tag (present on ≈0.4% of the available sites) and the linker are shown. (*B*) Flowchart of the number of samples and technical replicate runs used for screening OBOC DEL for Zika-specific candidates. The PICCOs library was screened in a stepwise manner using two well-characterized serum panels from documented ZIKV- and DENV-infected patients. (*C*) Illustration of the two-color screening procedure. The OBOC DEL is mixed with appropriate sera panels (Zika-immune sera versus flavivirus-naive sera, and dengue-acute versus -convalescent sera) at a fivefold redundancy, and positive beads are sorted out and collected for deep sequence of DNA-encoding tags.

We screened an OBOC DEL of PICCOs to identify ligands that recognize IgG antibodies from ZIKV-immune individuals. The library consisted of 508,032 unique molecular structures that were constructed by encoded split and pool solid-phase synthesis on 10 µm TentaGel beads carrying an invariant linker (17, 18) using published protocols (13–15). The synthetic workflow that was employed to construct the library is shown schematically in Fig. 1*A*. Each PICCO featured chemical diversity at six positions (R1 to R6). Positions R1 and R5 were derived from diverse chloroacids. Positions R2 and R6 were derived from amine building blocks. Position R3 was derived from Fmoc-protected amino acids that also contained an azide functionality and position R4 was derived from diverse aldehydes (*SI Appendix*, Figs. S1 and S2).

**Fig. 2.**
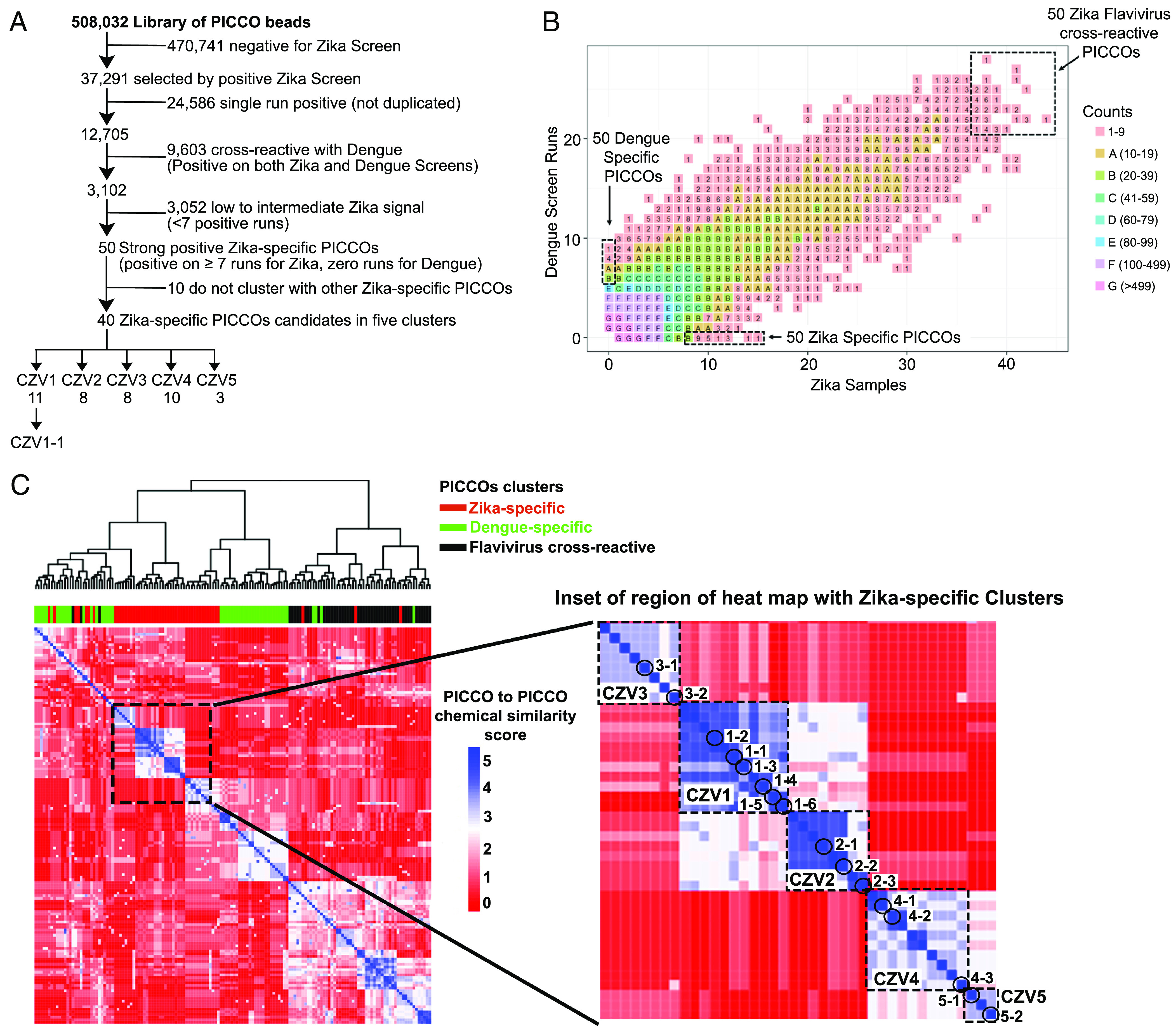
Hierarchical clustering of Zika-specific PICCOs based on chemical sequence identity. (*A*) Flowchart illustrating the down selection of Zika-specific reactive PICCOs identified during the screening procedure. (*B*) Distribution of PICCOs in terms of the number of sample hits. (*C*) Heatmap showing the chemical sequence similarity clustering of the screening candidate PICCOs. Five Zika-specific clusters encompassing 40 unique PICCO candidates were identified.

To identify ligands that bind specifically to anti-ZIKV IgG antibodies but not to IgG antibodies induced by infection with the closely related DENVs, the PICCOs library was screened in a stepwise manner using two well-characterized serum panels from documented ZIKV-infected patients (*SI Appendix*, Table S1) and DENV-infected patients (*SI Appendix*, Table S2). For detection and exclusion of dengue cross-reactive IgG antibodies, matched acute and convalescent sera were available from 16 dengue-infected patients. Matched acute and convalescent sera were pooled to form five matched pools of 2 to 4 patients each for flow cytometry testing (Fig. 1*B*). For Zika-specific IgG antibody detection, no acute sera were available, so highly characterized ZIKV-specific sera from five patients were tested against flavivirus-negative sera (negative for ZIKV and DENV antibodies) (*SI Appendix*, Table S3) (19). For the ZIKV screens, ten pools of two ZIKV-infected patients each were tested against three pools of flavivirus-negative sera (Fig. 1*B*). Positive PICCO-beads were identified and sorted out by two-color screen strategies (Zika-immune sera versus flavivirus-naive sera, and dengue-acute versus dengue convalescent sera; Fig. 1*C*). Because screening of bead-displayed libraries can have a significant false positive rate, we screened several copies of the library and only carried forward those structures that were selected as positive on multiple beads. We have shown previously that these “redundant hits” are usually bona fide ligands to the target antibody while “singletons” are usually false positives (13). Six replicate flow cytometric runs were performed for each of the five matched acute and convalescent dengue-infected serum pools, and two replicates were performed for each of the 30 combinations of the 10 unmatched Zika pools versus 3 naive serum pools (Fig. 1*B*). Fivefold redundancy of each PICCO, for a total of 2.5 million beads, was mixed with the labeled sera to allow for the detection of repeated PICCO molecular “hits” on each run. Each DNA-encoding Zika hit identified was then amplified, sequenced, and decoded using NGS to generate a list of putative PICCO ligands for anti-Zika IgG antibodies. We initially selected 37,291 unique PICCOs from the Zika antibody screens (Fig. 2*A*). PICCOs that were positive on a single run across multiple replicate runs were considered false positives (n = 24,586). After excluding PICCOS that were positive on both Zika and dengue antibody screens (n = 9,603), a total of 3,102 unique PICCOs (0.6% of the starting library of 508K PICCOs) were considered legitimate reactive Zika PICCO candidates. Because we are seeking truly Zika-specific PICCOs for use in serological assays, we focused on Zika-candidate PICCOs that reacted in at least seven Zika screen runs but zero dengue screen runs (n = 50). As expected, there was a considerable overlap of dengue-reactive and Zika-reactive PICCOs (Fig. 2*B*). This finding of PICCOs that bound with anti-flavivirus serum in two completely different experiments (dengue, and Zika) is evidence that these PICCOs are presumed to react with Zika/ dengue cross-reactive IgG antibodies and are likely to be surrogates for antigens with broad cross-reactivity across the flaviviruses.

### Hierarchical Clustering Reveals Clusters of ZIKV-Specific PICCOs as High-Priority Chemotype Candidates.

NGS analysis of the PICCO-displaying beads selected by FACS allowed for the generation of lists of the corresponding chemical structures (20). From the starting library of over 500,000 possible chemical structures, we selected 150 PICCOs of special interest based on their antibody binding patterns (*SI Appendix*, Table S4). Zika-only were those 50 PICCOs with multiple hits (more than seven runs) with Zika-positive sera but no hits with dengue positive sera; “dengue only” were those 50 PICCOS with multiple hits with dengue positive sera (more than five runs) but no hits with Zika-positive sera; and “flavivirus cross-reactive” were those 50 PICCOs with multiple hits with both Zika-positive sera and dengue positive sera (*SI Appendix*, Table S4). These structures were then subjected to a clustering analysis based on their degree of structural conservation at positions R1 through R6. Nearest neighbor hierarchical clustering was used to generate tree relationships and structure-based identity score heatmaps. A PICCO cluster was defined as multiple PICCOs that shared identical subunits at four or five of the six variable positions (Fig. 1*A*). Five PICCO clusters were identified that encompassed 40 of the 50 PICCOs of interest (Fig. 2*A*). Over 4,000 distinct Zika and dengue cross-reactive PICCOs were detected in more than seven runs, while 2,220 distinct Zika and dengue cross-reactive PICCOs were detected in more than 15 runs (*SI Appendix*, Fig. S3). For the top 50 Zika-specific PICCO candidates, only one unique PICCO compound was detected in a maximum of 15 runs (CZV1-1). We found five clusters of chemically similar Zika-only PICCOs, three clusters of dengue-only PICCOs, and eight clusters of flavivirus cross-reactive PICCOs (Fig. 2*C*). Of these five Zika-only clusters, the average cluster size was 8 PICCOs. All five PICCO unique compound’s identity clusters were internally homogeneous regarding their antibody binding properties, even though this was not a criterion for clustering and clustered exclusively with other chemically similar Zika-only PICCOs. The five Zika clusters were named to reflect the mean number of runs in which each distinct PICCO was detected (*SI Appendix*, Table S5). In the heat map, reading from the upper left down toward the lower right, the five Zika clusters in order are designated as clusters Zika 3 (CZV3), Zika 1 (CZV1), Zika 2 (CZV2), Zika 4 (CZV4), and Zika 5 (CZV5). This clear demonstration that PICCOs with chemical structure similarities also showed antibody binding similarities provides internal validation for our selection process. This clustering pattern suggests that some variability of PICCO chemical structure is permitted around an antibody-binding target without losing binding specificity. Altogether 40 of the 50 PICCOs of special interest clearly clustered with other similar PICCOs. The remaining 10 PICCOs, despite having been selected for multihit binding specificity, did not chemically cluster with other PICCOs and were deemed of lower priority in these studies.

### Hit Validation and Characterization of the Antibody-Binding “Pharmacophore”.

**Fig. 3.**
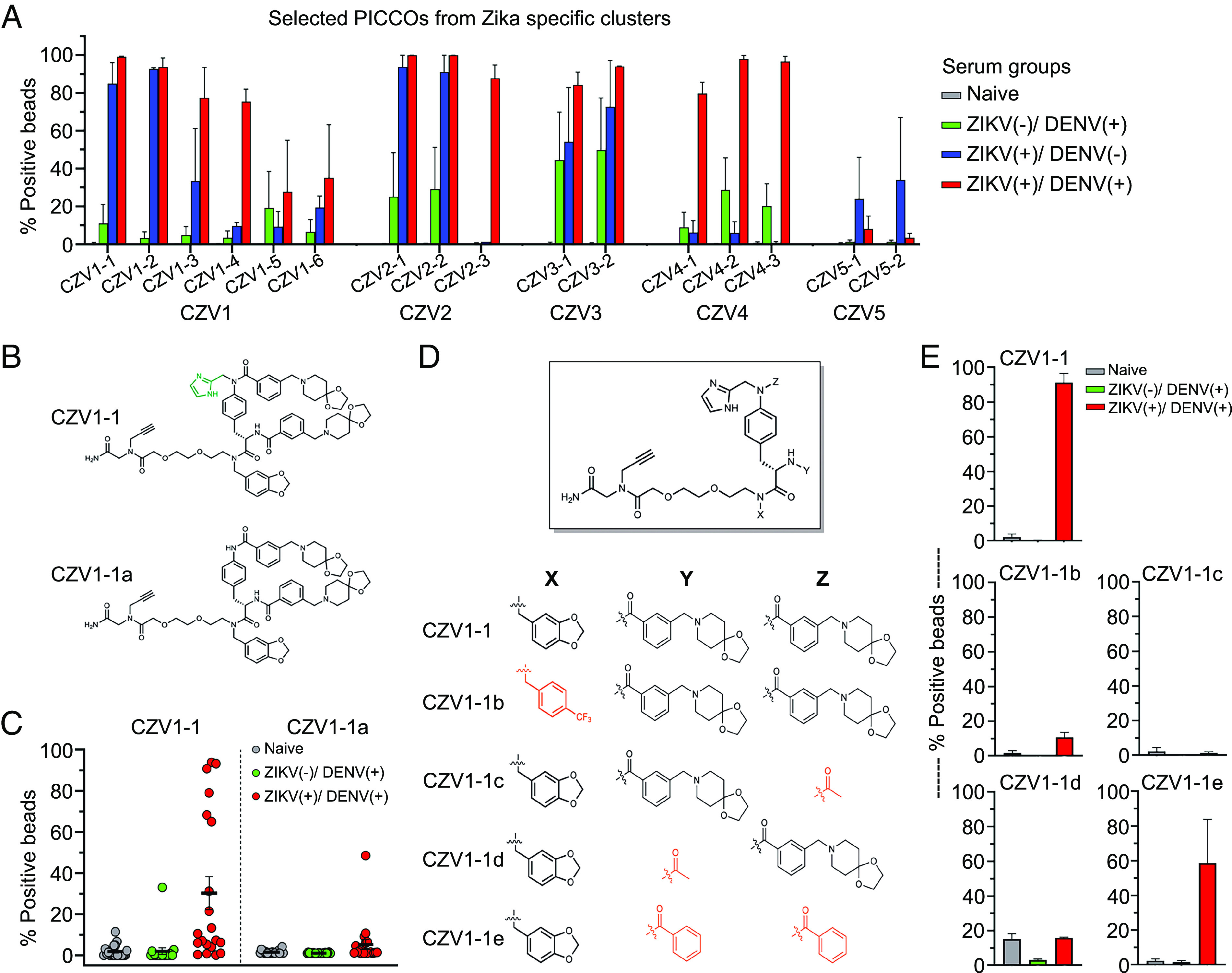
Validation and characterization of Zika-specific PICCO candidates. (*A*) Comparison of antibody specificity profiles of Zika-specific PICCOs from within the same chemical sequence cluster versus distinct clusters. (*B*) Anticipated structure of CZV1-1 and a “des-aldehyde” side product CZV1-1a and (*C*) the antibody binding profile against CZV1-1 and CZV1-1a. (*D*) Derivatives of CZV1-1 were synthesized to acquire additional information on how the ligand interacts with Zika-specific antibodies. (*E*) Binding profile of Zika-specific antibodies to CZV1-1 derivatives.

Following the clustering analysis, sixteen Zika-candidate diagnostic PICCOs from clear Zika-only clusters were selected for further patient-specific binding validation experiments. In addition to the Zika PICCOs, seven random, nonreactive PICCOs were included as negative controls. Each Zika-candidate PICCO was resynthesized in bulk on-resin without the DNA-encoding tag using 10µm TentaGel beads for further serum binding validation experiments. The validation serum panels included 1) two pools of three to four serum each from Zika IgG positive, dengue-IgG negative patients; 2) four pools of three serum each from dengue-IgG positive, Zika IgG negative patients; and 3) two pools of three serum each from patients positive for both Zika and dengue-IgG antibodies (*SI Appendix*, Table S6). The three pools of three samples each from *Flavivirus-*naive patients (IgG negative for Zika and dengue) were the same used for the screening step (*SI Appendix*, Table S3). Single-color FACS-based validation experiments were performed by incubating each pooled sera group with the selected Zika-candidate PICCOs. PICCOs and pooled sera were tested randomly to blind operators from ligand and sera specificity. Histograms were plotted and PICCOs were confirmed as positive by a shift to the right of distributions of the serum pools in comparison to blank control (bead without sera) and the percentage of positive beads was then calculated. All sixteen Zika PICCOs candidates were seroreactive: Six showed strong Zika specificity and no reactivity with dengue samples, five showed marginal reactivity, and five showed some level of unwanted cross-reactivity with dengue samples. The comparison of the antibody specificity profiles of PICCOs from within the same chemical sequence cluster versus different clusters reveals that PICCOs with similar chemical sequence structures have similar binding specificity profiles (Fig. 3*A*). Sera groups responded minimally to the set of negative controls PICCOs analyzed.

Based on these validation studies, we selected PICCO CZV1-1 as the most promising ligand for ZIKV-specific antibodies. CZV1-1 was resynthesized on-resin without the DNA-encoding tag on TentaGel beads containing a RAM linker, allowing the compound to be released from the resin and characterized by liquid chromatography/mass spectrometry. This is important because in the high-throughput library synthesis, unexpected events sometimes occur, and it is possible that a “side product” may be the active compound rather than the structure predicted by the DNA sequence. Indeed, using the same conditions that had been employed to construct the library, we found that the reductive amination reaction used to add the formyl imidazole unit (R4 in CZV1-1) to the resin was inefficient and the anticipated structure (CZV1-1) was only a minor product. This observation strongly suggested that the library beads presumed to display CZV1-1 displayed mostly a “des-aldehyde” analog, along with a small amount of CZV1-1. This called into question whether the imidazole unit was indeed important for binding the Zika antibodies. We developed improved conditions for this reductive amination that allowed the synthesis of pure CZV1-1 on-resin. When 10 µm TentaGel beads displaying bona fide CZV1-1 were employed in an antibody-binding assay using serum from Zika-infected patients, they robustly retained IgG from the sample. In contrast, beads displaying the des-aldehyde side product CZV1-1a displayed no significant IgG binding (Fig. 3 *B* and *C*). Samples from Zika-only patients (Zika-positive, dengue negative) were not included in this analysis due to limited serum availability. These data show that the imidazole group derived from the reductive amination at step R4 is essential for antibody recognition.

Several other derivatives of CZV1-1 were synthesized and assessed for their ability to bind Zika-specific antibodies to acquire additional information on how the ligand interacts with the antibody. We found that when an electron-poor aromatic ring was substituted for the electron-rich piperonyl ring at position R2 (CZV1-1b), binding was decreased by about 10-fold. Replacement of either one of the chloroacids + amine units at positions R5 (CZV1-1c) and R6 (CZV1-1d) with an acetyl group resulted in a complete and a 10-fold reduction in binding, respectively (Fig. 3 *D* and *E*). To determine whether this was largely a requirement of the chloro acid–derived R5 unit or the amine-derived R6 unit, we also synthesized compound CZV1-1e, in which a benzoic acid was placed at each position. In other words, the R5 unit was present but not the R6 unit. Strikingly, only a slight reduction in antibody retention was observed, arguing that the aromatic rings at each of the R5 positions are important for binding, but the amine-derived units at R6 are not. These data, which show a clear structure–activity relationship pattern, strongly support the idea that CZV1-1 is a selective ligand for the ZIKV-specific antibodies.

### CZV1-1 Discriminates between Zika and Other Flaviviruses Infections.

Next, the antibody binding properties of CZV1-1 were further examined using a larger set of serum samples (n = 226) with a broad repertoire of flavivirus immune reactivity. Binding to CZV1-1 was assessed for each serum individually using a single-color FACS-based assay and each serum was tested blindly without previous knowledge of the patient serostatus. The percentage of positive CZV1-1 beads was used for discriminating between Zika-positive and negative samples. The receiver operating characteristic curve showed that a threshold of 11.3% positive beads correctly classified patients as Zika- immune or not among all samples tested with a sensitivity and specificity of 85.3% (95% CI: 69.8 to 93.5) and 98.4% (95% CI: 95.5 to 99.6), respectively (Table 1 and *SI Appendix*, Fig. S4). For the DENV-immune, ZIKV-naive individuals, only 1.6% of the samples showed reactivity with CZV1-1 beads with a percentage of positive beads above the cutoff. Among the DENV-immune individuals, the sensitivity and specificity of assays were 82.7% (95% CI: 65.4 to 92.4) and 98.3% (95% CI: 94.2 to 99.7), respectively (Table 1 and *SI Appendix*, Fig. S4). For this study, we tested only one PICCO, CZV1-1, in detail for its diagnostic properties.

**Table 1.** Specificity and sensitivity analysis of the CZV1-1 assay on a large panel (n = 226) of ZIKV-naive and ZIKV-immune samples

Results with conventional assays				Binding to CZV1-1		
*ZIKV*	*DENV*	YFV^*^	CHIKV	*Sera tested*	*Sera positive*	%Sera positive
−	−	−	−	61	1	1.6%
−	+	−	−	55	2	3.6%
−	−	+	−	5	0	0%
−	−	−	+	5	0	0%
Total ZIKV negatives				192	3	1.6%
+	−	−	−	5	5	100%
+	+	−	−	29	24	82.7%
Total ZIKV positives				34	29	85.3%

ZIKV: Zika virus, DENV: dengue viruses, CHIKV: chikungunya virus, YFV: yellow fever virus.

^*^Samples from YFV vaccinees.

### Identification of the Native Antigen Recognized by CZV1-1-Binding Antibodies.

**Fig. 4.**
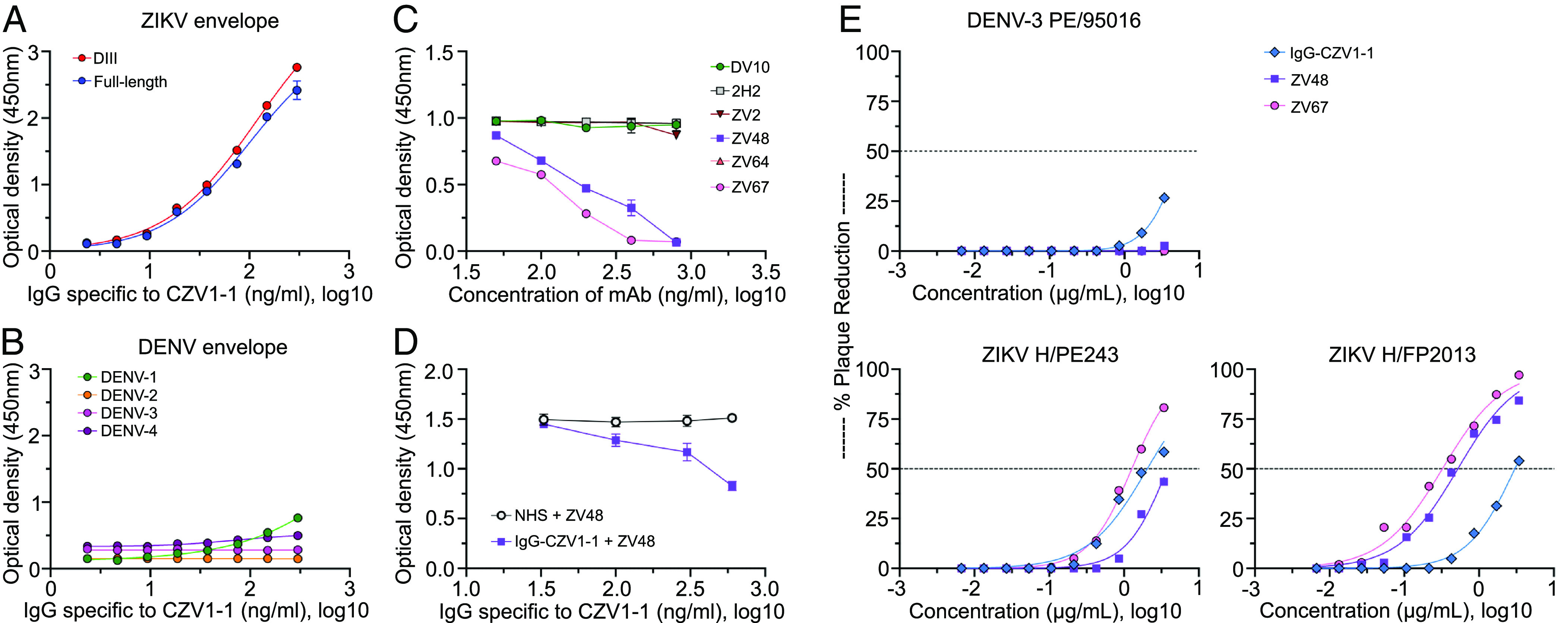
Identification and characterization of the native antigen recognized by CZV1-1-binding antibodies. Binding profile of the purified IgG specific to CZV1-1 against (*A*) ZIKV envelope protein (full-length and DIII) and (*B*) DENV1-4 envelope proteins. (*C*) Blockage of binding experiments of CZV1-1 interaction with ZIKV envelope by competition with known mouse monoclonal ZIKV antibodies. (*D*) Reverse blocking experiments to assess the competition of the mAbs for binding of IgG specific to CZV1-1 to the ZIKV envelope. NHS, normal human serum. (*E*) Neutralization of ZIKVs (French Polynesia and Brazilian strains) but not DENV-3 by the purified IgG specific to CZV1-1.

We next focused on identifying the native antigen recognized by the IgG antibodies that bind specifically to CZV1-1. First, CZV1-1 was covalently attached to an agarose column, and pooled ZIKV-immune sera were used to enrich for IgG that binds specifically to CZV1-1. Two rounds of purification were performed using a pool of serum samples from ZIKV-immune individuals that had shown strong reactivity to CZV1-1 in the FACS-based assay. Pooled serum was passed over the column with immobilized CZV1-1 and, following washing, bound IgG was released from the resin using a gentle antigen/antibody elution buffer (pH 6.6). The concentration of IgG following buffer exchange was determined by ELISA. The fraction of IgG specific to CZV1-1 isolated corresponded to ~0.3% of the total IgG present in the initial pooled sera used for affinity purification. FACS-based assay confirmed the binding of this purified CZV1-1-specific IgG to CZV1-1 PICCO-beads and revealed that the enriched IgG had an ~600-fold increase in specific binding when compared to the initial pooled ZIKV-immune sera. We then assessed the binding of CZV1-1-specific IgG against an array of ZIKV and DENV structural and nonstructural antigens. We found that CZV1-1-specific IgG binds strongly to full-length ZIKV envelope protein as well as to domain three (DIII) of the ZIKV envelope, with 50% binding concentrations of 100ng/ml and 126ng/ml, respectively (Fig. 4*A*). CZV1-1-specific IgG showed no or weak reactivity to the full-length envelope of DENV1-4 (Fig. 4*B*) and no reactivity to ZIKV and DENV1-4 NS1 (*SI Appendix*, Fig. S5).

To gain further insights into the natural epitope target corresponding to CZV1-1, we performed antibody competition assays against several mAbs known to bind spatially distinct epitopes on DIII of ZIKV envelope (mouse mAbs ZV2, ZV48, ZV64, and ZV67) (12). Two mAbs that bind to DENV envelope DIII (DV10) and prM (2H2) were also included as controls. IgG from flavivirus-naive human subjects was included as a source of negative control sera. We first assessed the binding of CZV1-1-specific IgG and the mAbs directly to the ZIKV envelope DIII protein. We found that CZV1-1-specific IgG, and two of the four ZIKV mAbs (ZV48 and ZV67), were able to bind with high potency to the ZIKV envelope DIII protein used in our assays (*SI Appendix*, Fig. S6). We next compared the abilities of these different mAbs to block the binding of CZV1-1-specific IgG to the ZIKV envelope DIII captured on an ELISA plate. We found that CZV1-1-specific IgG exhibited substantial competition for binding with ZV48 and ZV67, as 100 ng of these mAbs blocked 50% binding of 100 ng/mL of the IgG specific to CZV1-1 (Fig. 4*C*). We next performed reverse blocking experiments to assess the competition of the mAbs for binding of IgG specific to CZV1-1. We found that CZV1-1-specific IgG competes for binding with ZV48 (Fig. 4*D*) but not ZV67 (*SI Appendix*, Fig. S7) suggesting that IgG against CZV1-1 sterically hinders access of ZV67 mAb to its epitope target when coated in the solid-phase. In addition, ZV67 has the highest affinity to its epitope when compared to ZV48, which might have contributed to the lack of competition with IgG against CZV1-1 in the reverse blocking experiment. More importantly, our blocking experiments suggest that CZV1-1 is a surrogate for the C- C’ loop epitope which projects away from the “sandwich” core of ZIKV envelope DIII.

Because the ZIKV-specific mAbs used in these experiments not only recognize antigen determinants in the DIII of the ZIKV envelope but are also known to have neutralization properties, we next performed neutralization assays using multiple ZIKV strains. One dengue strain isolated in Brazil (DENV-3, 95016/BR-PE/02) was included as a control for the assay. Remarkably, we found that CZV1-1-specific IgG neutralizes the Brazilian ZIKV strain (PE243) with a potency similar to that of monoclonal antibody ZV67 (IC50: 2.05 versus 1.27 μg/mL) and better than ZV48 (IC50: 2.05 versus 3.8 μg/mL). CZV1-1-specific IgG also neutralized the ZIKV French Polynesia strain (H/PF2013) but with lower potency than ZV48 and ZV67 (Fig. 4*E*). We speculate that because the CZV1-1-specific IgG antibodies used in these studies were collected from Brazilians who were infected with ZIKV, this may be the reason why they neutralized the Brazilian strain better than the French Polynesian strain. The number of Zika strains that could be tested here was limited by the quantity of our purified human CZV1-1-specific IgG.

**Fig. 5.**
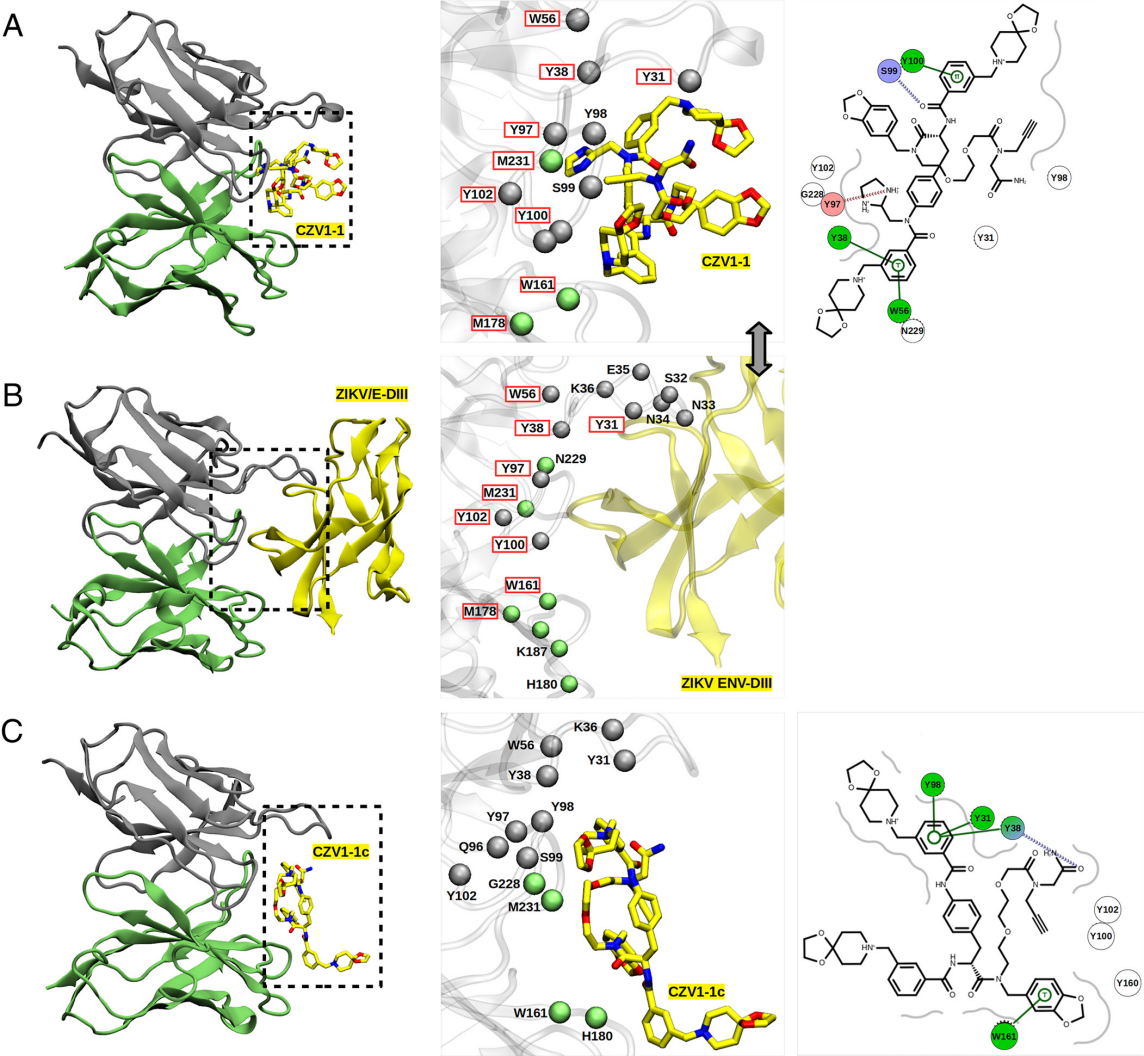
Binding mode and intermolecular interactions of the ZV48/CZV1-1 complexes. Displayed molecular representations correspond to the last frame of MD simulation for the ZV48/CZV1-1 (*A*), ZV48/ENV-DIII (*B*), and ZV48/CZV1-1c (*C*). The light and heavy chains of the mAbs are represented in silver and green, respectively; spheres represent the position of α-carbon atoms of the backbone. Residues highlighted in red represent common interactions of the ligands and ENV-DIII with the mAb. The 3D model of the CZV1-1 adducts 1 and 1c are shown using a stick model, and atoms are color-coded as yellow for carbon, blue for nitrogen, and red for oxygen (hydrogen atoms were omitted for clarity). The following amino acids of ZV48 make contact with both ZIKV/E-DIII and the CZV1-1 adduct: Y31, Y38, W56, Y97, Y100, Y102, W161, M178, and M231.

Molecular docking simulations suggested that CZV1-1 binds to mAb ZV48 with higher affinity (*SI Appendix*, Table S7). To further evaluate the flexibility and intermolecular interactions of the mAbs and CZV1-1 complexes, molecular dynamics (MD) simulations were performed. MD trajectory analyses showed that CZV1-1 exhibits a high rate of native contacts and hydrogen bonds with mAb ZV48 than other mAbs (*SI Appendix*, Figs. S8 and S9). Furthermore, CZV1-1 shares a greater number of common interactions with the ZIKV DIII envelope subunit when bound to mAb ZV48 (Fig. 5 *A* and *B*), as compared to CZV1-1c that was used as a control for the MD simulations (Fig. 5*C*). These interactions include ZV48 residues Y31, Y38, W56, Y97, Y100, Y102, W161, M178, and M231 (*SI Appendix*, Fig. S8). It is worth noting a network of π-stacking interactions between residues Y101, Y39, and W57 and the aromatic rings of CZV1-1; a hydrogen bond with the side chain of the amino acid S100, and a salt-bridge with Y98 (Fig. 5*A*). In contrast, molecular docking showed a low affinity of CZV1-1c (negative control) to mAb ZV48 (*SI Appendix*, Table S7). These results are confirmed by metadynamics simulations of the CZV1-1 and CZV1-1c adducts and the mAbs ZV48 and ZV67 (*SI Appendix*, Fig. S10).

## Discussion

In this study, we extended our prior experience in screening combinatorial libraries of small synthetic molecules (13–15) to efficiently identify a small synthetic molecule CZV1-1 that binds specifically to IgG present in serum from Zika-immune persons, but not in serum from dengue-immune persons. Our PICCO library design was antigenically agnostic—one that does not require foreknowledge of the pathogen structure or sequences—in that there was nothing about the half million small molecules that should have preferentially presented ZIKV antigenic mimics. Nonetheless, we were able to identify and resynthesize a ZIKV molecular mimic that has real potential as a diagnostic reagent to distinguish between natural infections caused by the two closely related ZIKV and DENVs. Our CZV1-1 assay has a remarkably superior specificity when compared to the other protein-based assays aiming at detecting ZIKV-specific IgG. The diagnostic sensitivity of CZV1-1 alone was above 80%. We acknowledge that there are other protein-based assays with better sensitivity reported (21, 22). However, this sensitivity could be further improved by additional structure–activity research to optimize the antibody binding properties of CZV1-1. It is also possible that further development of PICCOs from other structural clusters that are distinct from CZV1-1 identified in this study could lead to the development of a multi-PICCO assay format. Further characterizations of the binding properties against a wide array of other flaviviruses should also be done. If CZV1-1-based assays continue to show favorable diagnostic properties, they could have considerable value in high-risk situations like ZIKV infections in pregnancy as a risk assessment tool in the prenatal screening of pregnant women. In addition, CZV1-1 might be an alternative tool for PRNT (plaque reduction neutralization test) assays as a confirmatory test to differentiate antibodies to related viruses. This approach for the identification of specific small-molecule biomarkers of viral infection and immunity, shown here in the specific case of Zika, can potentially be applied to any virus. Indeed, the general strategy of identification of antibody biomarkers by comparing the PICCO binding profile of IgG in serum specimens from patients with, versus those without, a disease might also be applicable to noninfectious diseases such as cancers, autoimmune diseases, and other chronic conditions.

A critical component of this study was our ability to construct “gold-plated” panels of Zika-immune and dengue-immune human sera that were carefully characterized by conventional ELISA and neutralization assays. Although we were unable to determine the timing of sample collection after infection for the Zika-immune individuals used for our initial screens, all serum samples were confirmed to show no reactivity with dengue antigens in ELISA and neutralization assays, confirming that samples were highly ZIKV-immune specific. Then, in our validation and evaluation experiments, we showed that samples from individuals with immunity to both Zika and dengue infections still retained IgG that bound specifically to CZV1-1. This is important since most individuals living in ZIKV-endemic areas have background exposure to multiple flaviviruses.

DNA-encoding of the PICCO-displaying beads enabled NGS-based analysis which provided rapid elucidation of the chemical structures identified during the screening steps. Our clustering analysis provided insights into the structural similarities and features important for Zika-specific IgG binding to these molecules. Our data showing similar IgG binding profiles within hit candidates selected from the ZIKV-specific clusters revealed that a larger set of potential, analogous small-molecule candidates could also be used for improving diagnostic accuracy. Affinity purification experiments yielded concentrated CZV1-1-specific IgG antibodies. Subsequent binding competition experiments revealed that the purified CZV1-1-specific IgG competed with mAbs directed to the DIII of ZIKV envelope protein, indicating that CZV1-1 epitope mimetic was within or overlapped with DIII. More specifically, CZV1-1-specific IgG competed strongly with monoclonal antibody ZV48 suggesting that CZV1-1 mimics a cryptic epitope in the C-C’ loop arranged within the sandwich core of DIII (12). The fact that IgG against an epitope that is not predicted to be accessible on the mature ZIKV virion can be detected in samples from immune individuals further indicates that viral breathing likely allows the exposure of the C-C’ loop during ZIKV infections in vivo (23–25). Purified CZV1-1-specific IgG has neutralization activity against at least two ZIKV strains, which suggests the potential for CZV1-1 to be used as a vaccine immunogen. A previous study has shown that peptoids (N-substituted oligoglycines) conjugated with a carrier protein induce a robust immune anti-peptoid response in mice (26). Although results from this study support the notion that fully synthetic, nonbiological, small molecules have the potential to be used as vaccines, further experimental data to confirm that CZV1-1 would generate the same type and quality of antibody as ZV48 when used as a vaccine is still needed.

In summary, by comparing the binding profiles of Zika and dengue human antibodies to a large random library of small molecules, we identified, produced, and characterized a small nonbiological synthetic molecular mimic (CZV1-1) of a dominant Zika envelope protein-neutralizing epitope. Importantly, the small molecule CZV1-1 corresponds to a conformational epitope that would be difficult to synthesize from knowledge of the envelope protein structure. We have shown here that the CZV1-1 PICCO molecule can serve as a biomarker on which to base diagnostic assays for evidence of prior Zika infection. Because it is a mimic of a neutralizing epitope, it may also serve as a biomarker of immunity to Zika. Finally, the general strategy we employed here to identify and characterize a small-molecule mimic of a virus-neutralizing epitope for Zika can probably be used to find small-molecule mimics of important epitopes for a wide range of other infectious and noninfectious diseases.

## Materials and Methods

### Library Synthesis and Screening and NGS.

The DEL employed for this study was constructed by encoded split and pool solid-phase synthesis on 10 µm TentaGel beads carrying the invariant linker (Fig. 1*A*). A total of 2.5 million beads per tube were used for FACS-based library screening to account for a fivefold redundancy of the library. Pooled sera were prepared at a concentration of 50 mg/mL of total protein and labeled using PE-conjugated or Alexa Fluor 647–conjugated goat anti-human IgG. Labeled sera were then collected and incubated with preblocked DEL beads. Following overnight incubation, plates were washed and beads were resuspended in phosphate-buffered saline (PBS) and sonicated before FACS acquisition and sorting in a FACS Aria III. Sorted beads were transferred to PCR tubes for onbead PCR amplification of the DNA string that corresponds to the chemical on the bead. Amplified DNA products were analyzed using NGS, and FASTQ files were received from the IonTorrent IonProton instrument. IonTorrent barcodes were used to separate information from individual screening campaigns as different files. DNA sequence deconvolution allowed analysis of the chemical structure on the beads. Full details are provided in *SI Appendix*.

### Serum Sample Panels.

For PICCOs library screening, the serum panel from Zika-infected patients consisted of PCR-confirmed Zika infections of dengue-naive individuals collected from several clinical-epidemiological studies carried out in Recife, Northeast Brazil (27–31). These samples were serologically characterized as Zika reactive only by binding IgM and IgG assays for dengue, Zika, and Chikungunya and by neutralization assays for dengue and Zika (*SI Appendix*, Table S1). The dengue panel consisted of serial samples from patients with confirmed (by PCR, IgM, and IgG and neutralization assays) primary dengue infections and divided into two groups: a) acute samples collected no more than 8 d from the start of the symptoms and confirmed as dengue-IgG negative by ELISA, and b) early (<3 mo) and late convalescent samples confirmed as anti-dengue-IgG positive. Dengue samples were from two natural history cohorts carried out in Northeast Brazil before Zika introduction in the area (2004 to 2009) (*SI Appendix*, Table S2) (32, 33). For hit validation, pooled samples from Zika IgG positive, dengue-IgG negative patients (n = 2), dengue-IgG positive, Zika IgG negative patients (n = 4), Zika and dengue-IgG positive patients (n = 2), and from *Flavivirus-*naive patients (n = 3) were included. For the evaluation of the diagnostic performance of the selected PICCO candidate, and antigen surrogate identification, a larger set of well-characterized serum samples (n = 226) was included (27, 32, 34, 35). Zika-immune patients included 34 DENV-naive and 29 DENV-immune samples. Among the ZIKV-immune patients, eight were virologically confirmed ZIKV cases (all previously DENV-immune), and nine were ZIKV cases confirmed by IgM and IgG seroconversion between acute and convalescent samples. The remaining ZIKV-immune samples were positive for IgG and PRNT for ZIKV. Samples from ZIKV-naive individuals included DENV-naive (n = 61), DENV-naive, YFV vaccinees (n = 5), DENV-naive, CHIKV-immune (n = 5), DENV-immune, multiple serotype exposures (n = 55), and DENV-immune, CHIKV-immune (n = 66) patients. Full details are provided in *SI Appendix*.

Ethical approval for the clinical and epidemiological studies was obtained from the institutional review boards of the Brazilian Ministry of Health, Aggeu Magalhaes Institute, Oswaldo Cruz Foundation, and the Pan American Health Organization. All participants included provided informed consent. For use in the current study, all human samples were deidentified. The University of Pittsburgh Institutional Review Board determined that its approval was not required because participating University of Pittsburgh investigators were not involved in human subject research.

### Hierarchical Clustering Analysis.

This analysis was carried out to determine whether two or more high-hit PICCOs were similar in structure. We assigned a probability of similarity for each of the six chemical components based on the number of alternative components at that site. For positions one through six, the number of chemical component synthesis options at each position was, respectively, 2, 6, 6, 81, 7, and 13. To compare the similarity or dissimilarity of any two PICCOs in pairwise matches, we calculated and expressed this similarity as the empirical probability of observing the identical chemical component at each of the six chemical oligomer positions. To do this, we first calculated the frequency distributions of the chemical structures at each of the six oligomer positions from among 54,853 randomly selected PICCOs. This generated empirical values for the probability of chemical identify at each of the six oligomeric positions of 0.501,0.169, 0.203, 0.0141, 0.192, 0.102, respectively. The distance was defined with log10 of the probability with the range 0 (completely different) to of −5.32 (identical). For example, the probability that two PICCOs would have an identical chemical structure at positions two, three, four, five, and six is roughly log10 (0.169*0.203*0.0141 *0.192*0.102) = −5.02. The similarity or dissimilarity between PICCOs is displayed in Fig. 2*C* as the absolute value of this log distance, with red colors (<3) signifying dissimilar PICCO oligomer chemical structures and blue colors (> 3) signifying similar PICCO oligomer chemical structures. The hierarchical clustering method used was Ward’s minimum variance method, to generate compact, spherical clusters. The R code function “hclust()” with method=”ward.D2” was used in generating the heatmap. Chemical structure clusters were defined as contiguous groups with similarity probability scores of −4 or lower.

### Affinity Purification of IgG Antibody from the Serum.

Antibodies that bind CZV1-1 were enriched from a pool of ZIKV-immune sera by affinity purification. CZV1-1 was covalently immobilized to a SulfoLinkTM affinity column (ThermoFisher) following the manufacturer’s protocol. Undiluted, pooled ZIKV-immune sera was incubated to the column overnight at 4 °C. The column was then washed, and the bound IgG was eluted using Gentle Ag/Ab elution buffer pH 6.6 (ThermoFisher). The IgG was dialyzed overnight, and the sample was concentrated using centrifugal concentrators. Total IgG levels were quantified using a sandwich ELISA (19). Full details are provided in *SI Appendix*.

### Binding IgG ELISA Assays.

To assess the binding of the purified IgG-CZV1 to different ZIKV and DENV antigens, high-binding, half-area 96-well flat-bottom polystyrene plates (Corning) were coated with the following recombinant proteins (at 2 µg/mL) or cell extracts in carbonate/bicarbonate buffer overnight at 4 °C: ZIKV or DENV envelope protein (Sino Biologicals), ZIKV or DENV1-4 NS1 proteins (Native Antigen), ZIKV or DENV1-4 cell extracts, and ZIKV or DENV1-4 cell lysates. Plates were blocked with nonfat dry milk (NFDM; Bio-Rad) at 5% (w/v) in PBS for 1 h at room temperature. Then, purified IgG-CZV1 diluted in 5% (w/v) of NFDM in PBS were added to the plate at different concentrations and incubated for 1 h at room temperature. Plates were washed six times with Tween-20 at 0.5% in phosphate-buffered saline (PBS-T) and incubated for 1 h with horseradish peroxidase (HRP)-linked goat anti-human IgG (Jackson ImmunoResearch). After five washes with PBS-T, plates were incubated at room temperature for 20 min with HRP substrate, 3,3′,5,5″-tetramethylbenzidine (SeraCare), and the developing reaction was stopped by adding 1 N hydrochloric acid (Sigma). Optical densities at a wavelength of 450 nm (OD450 nm) were measured using SpectraMax Plus PC380 microplate spectrophotometer using SoftMax Pro software version 6.4 (Molecular Devices). Optical densities from blank wells were subtracted from all measurements before analysis.

### Blockade-of-Binding (BOB) ELISA Assays.

BOB assay measures the level of purified IgG specific to CZV1-1 that blocks the binding of highly specific mAbs to the ZIKV envelope. Briefly, high-binding, half-area 96-well flat-bottom polystyrene plates (Corning) were coated with 2 µg/mL of ZIKV or DENV recombinant envelope protein (Sino Biologicals) in carbonate/bicarbonate buffer overnight at 4 °C. Plates were blocked with NFDM (Bio-Rad) at 5% (w/v) in PBS for 1 h at room temperature. Then, purified IgG-CZV1 diluted in 5% (w/v) of NFDM in PBS were added to the plate at different concentrations (600, 300, 100, and 33 ng/mL). Plates were then incubated with IgG-CZV1 for 30, 45, and 55 min. A solution containing 200 ng/mL of ZIKV or DENV-specific mouse mAbs (ZV-2, ZV-67, ZV-64, ZV-48, DV-10, and Absolute antibody) was immediately pipetted on top of the IgG-CZV1 sample and incubated for 30, 15, and 5 min for a total incubation period of 60 min. Plates were washed with PBS-T and incubated with HRP-linked goat anti-human IgG (Jackson ImmunoResearch). For the reverse blocking experiments, mAbs diluted in 5% (w/v) of NFDM in PBS were added to the plate at different concentrations (800, 400, 200, 100, and 50 ng/mL) and were incubated for 30 min. A solution containing 200 ng/mL of purified IgG-CZV1 was immediately pipetted on top of the mAbs and incubated for an additional 30 min. Plates were washed with PBS-T and incubated with HRP-linked goat anti-mouse IgG (Jackson ImmunoResearch). After washing, plates were incubated with HRP substrate, 3, 3′, 5, 5″-tetramethylbenzidine (SeraCare), and the developing reaction was stopped by adding 1 N hydrochloric acid (Sigma). Optical densities at a wavelength of 450 nm (OD450 nm) were measured using SpectraMax Plus PC380 microplate spectrophotometer using SoftMax Pro software version 6.4 (Molecular Devices). Optical densities from blank wells were subtracted from all measurements before analysis.

### Neutralization Assays.

The PRNT was used to assess the neutralization activity of the purified IgG-CZV1 following a protocol described in detail elsewhere (36). The assay was carried out using the following virus strains: ZIKV strain *H. sapiens*/PE243/2015 (Brazil 2015), ZIKV strain *H. sapiens*/PF/2013 (French Polynesia, 2013), and DENV-3 *H. sapiens*/PE/02-95016 (Brazil 2002). The PRNT positivity was defined based on a 50% reduction in plaque counts (PRNT_50_), and neutralizing antibody titers were estimated using a four-parameter nonlinear regression.

### Molecular and Metadynamic Simulations.

The binding pose prediction of the CZV1-1 adducts was performed using a semiflexible approach coupled to the genetic algorithm of the GOLD software (37), considering the following x-ray structures as receptors: 5KVE, 5KVG, and 5VIC (9, 38). The x-ray structures 5KVE and 5KVG correspond to the ZIKV envelope protein complexed to the mAbs ZV48 and ZV67, respectively; and 5VIC corresponds to the envelope protein of DENV-1 complexed to neutralizing mAb Z004. The final structures from the MD simulations were used as a starting point for metadynamics calculations using Plumed version 2.0.1 (39) within NAMD version 2.13 (40). Full details are provided in *SI Appendix*.

## Supplementary Material

Appendix 01 (PDF)

## Data Availability

Anonymized NGS data have been deposited in GEO (GSE254062).
