# Supplementary material for: Identification and characterization of a nonbiological small-molecular mimic of a Zika virus conformational neutralizing epitope
Source: Proc Natl Acad Sci U S A. 2024 May 14;121(21):e2312755121. doi: 10.1073/pnas.2312755121 (PMC11127016; doi:10.1073/pnas.2312755121)
Supplement: Supplementary file 1 — Appendix 01 (PDF) [file pnas.2312755121.sapp.pdf]

Supporting Information for

Identification and characterization of a nonbiological small-molecular mimic of a Zika virus conformational neutralizing epitope.

Priscila M. S. Castanha, Patrick J. McEnaney, Yongseok Park, Anthea Bouwer, Elton J. F. Chaves, Roberto D. Lins, Nicholas G. Paciaroni, Paige Dickson, Graham Carlson, Marli T. Cordeiro, Tereza Magalhaes, Jodi Craigo, Ernesto T. A. Marques*, Thomas Kodadek*, Donald S. Burke*

*Corresponding author(s) e-mail address(es): marques@pitt.edu, kodadek@ufl.edu, donburke@pitt.edu

This PDF file includes:

Supporting text (Materials and Methods)
Data, Materials, and Software Availability
Figures S1 to S10
Tables S1 to S7
SI References

Supporting Information Text

Detailed Materials and Methods.

Library synthesis. The DNA-encoded library (DEL) employed for this study was constructed by encoded split and pool solid-phase synthesis on 10 µm TentaGel beads carrying the invariant linker (see Fig. 1A). The split and pool process results in the production of a one-bead-one compound library, i.e., each bead display many copies of a single compound, assuming perfect chemistry. The library was made in six cycles. First, a bead-displayed primary amine was acylated with diverse chloroacids. The chloride was then displaced with diverse amines. The resultant secondary amine was acylated with a Fmoc-protected amino acid also containing an azide moiety. The azide was reduced and subjected to a reductive amination reaction with diverse aldehydes. The Fmoc protecting group was then removed and the free amine was acylated with diverse chloroacids. The chloride was then displaced by diverse amines to complete the synthesis. This six-cycle OBOC DEL theoretically contains 508,032 unique compounds.

FACS-based PICCOs library screening. DNA-encoded library beads were pre-blocked in 1mL of blocking buffer for 1 hour at 4°C under rotation. Pooled sera were prepared at a concentration of 50mg/ml of total protein and labeled using PE-conjugated or Alexa fluor 647-conjugated goat-anti human IgG (Jackson ImmunoResearch) in tubes under rotation for 30min at room temperature. Following, excess PE- or Alexa fluor 647-conjugated antibodies were removed by incubation with human agarose IgG for 10 minutes in a filter plate followed by centrifugation. Labeled sera were then collected and incubated with DNA-encoded library beads overnight at 4°C. The next day, the plate was washed three times with 150µl PBS-0.1% Tween, with the plate shaking for 5 min in the cold room, between spins. Beads were then resuspended in PBS and sonicated before FACS acquisition and sorting in a FACS Aria III. After sorting, beads were transferred to PCR tubes for onbead PCR amplification of the DNA string that corresponds to the chemical on the bead. PCR-amplified products were run into polyacrylamide gels, then bands were isolated and subjected to DNA extraction using commercially available kits (Quiagen) and subjected to sequencing.

FACS-based binding assays. For the hit validation, evaluation of diagnostic performance, and antigen surrogate discovery, single-color FACS-based binding assays were performed. A total of 25,000 beads/well were pre-blocked using blocking buffer. Patient sera at 1:10 dilution was stained with PE-conjugated goat-anti-human IgG (Jackson ImmunoResearch). Excess PE-conjugated antibodies were removed by incubation with human agarose IgG for 10 minutes in a filter plate followed by centrifugation. Labeled sera was then added to the beads and the plate was incubated overnight in the cold room on a plate shaker. The next day, the plate was washed three times with PBS-0.1% Tween, with the plate shaking for 5 min in the cold room, between spins. After the last wash, beads were resuspended in PBS-T and transferred to FACS tubes. Samples were analyzed on LSR II or Fortessa.

Affinity-purification of IgG antibody from serum. Antibodies that bind CZV1-1 were enriched from a pool of ZIKV immune sera by affinity purification. CZV1-1 was covalently immobilized to a SulfoLinkTM affinity column (ThermoFisher) following the manufacturer's protocol. Ten mL of resin slurry was added to a plastic column and resin was settled by gravity-flow. 5mg of CZV1-1 cysteine conjugated was dissolved in coupling buffer (50mM Tris, 5mM EDTA, pH 8.5) and incubated for 45 minutes at room temperature. After washing in PBS containing 1M NaCl, unreacted iodoacetamide groups were quenched using a solution of 50mM L-Cysteine-HCl in coupling buffer for 45 minutes at room temperature, and then equilibrated into TBS. Undiluted, pooled ZIKV immune sera was incubated to the column and allowed to soak into resin bed for 10 minutes. Then, TBS was added to the column and serum antibodies were hybridized to the affinity column by incubating overnight at 4°C. The column was washed with TBS and the bound IgG was eluted using Gentle Ag/Ab elution buffer pH 6.6 (ThermoFisher). The IgG was dialyzed overnight in TBS and the sample was concentrated using centrifugal concentrators. Total IgG levels were quantified using a sandwich ELISA (1).

Next-generation sequencing (NGS). Each bead collected by FACS contained on its surface both a chemical ligand (PICCO) and a DNA string that encoded the chemical structure of that PICCO. The encoding DNAs on the beads were amplified by PCR to produce a soluble DNA pool, analyzed using NGS, and FASTQ files were received from the IonTorrent IonProton instrument. IonTorrent barcodes were used to separate information from individual screening campaigns as different files. DNA sequence deconvolution allowed analysis of the chemical structure on the beads. Each DNA string contained 10 unique encoding sites, separated by 3 bp distinct overhangs for successive ligations during molecule synthesis and in the analysis process for data pattern matching. Each encoding site consisted of an eight-nucleotide string, which were identified by their surrounding overhangs. To ensure the differentiation of multiple beads with the same chemical identity (and thus identical PICCO DNA codes), the first two of the 10 encoding sites were used as a 'bead-specific' barcode, which enabled differentiation of up to 100 separate beads with the same chemical structure. The last two encoding sites on the DNA identified the user of the data. The middle six encoding sites corresponded to the chemical synthesis steps. Data analysis consisted of a full-length molecule trimming, pattern matching each DNA string, and translating the 10-barcode DNA strings into a sequence of numbers that revealed the identity of each specific bead, the chemical structure, and the user. In the next step, we identified "hit" beads based on the number of NGS reads. By counting DNA reads with the same full molecule code string sequence we used a read count cutoff of a minimum of 30 reads to identify "true hit" beads. True hits with identical chemical structure codes but differing at the first two "bead specific" positions were used to identify and count multiple redundant bead hits for that chemical structure. The number of bead hits for each chemical structure was used as a measure of that chemical structure's recognition by patient antibodies.

Molecular simulations. The binding pose prediction of the CZV1-1 adducts was performed using a semi-flexible approach coupled to the genetic algorithm of the GOLD software (2), considering the following x-ray structures as receptors: 5KVE, 5KVG, and 5VIC (3, 4). The x-ray structures 5KVE and 5KVG correspond to the ZIKV envelope protein complexed to the mAbs ZV48 and ZV67, respectively; and 5VIC corresponds to the envelope protein of DENV-1 complexed to neutralizing mAb Z004. We then performed the following preparation steps: (i) water molecules were removed; (ii) resulting structures were aligned as a function of domain III of the ZIKV ENV (only backbone atoms were used); (iii) the center of mass (COM) was calculated considering residues within the protein-protein interface; (iv) ENV was removed. The COM information was used to center the grid box at the protein-protein interface. All residues within a cutoff radius of 15 Å from this point. The following parameters in the GOLD software were used: (i) autoscale equal to 2, which means that the genetic algorithm operates exhaustively; and (ii) the Astex Statistical Potential (ASP) scoring function was used to score the binding poses. Other parameters were kept in default mode. Molecular dynamics simulations were performed by starting from previously modeled coordinates for the mAbs ZV48, ZV67, and Z004 docked with CZV1 adducts 1 and 1c. The protein and ligand were parameterized with the AMBER force fields FF14SB (5) and GAFF (6). pKa calculations were performed with PDB2PQR (7) to evaluate the protonation states of each titratable residue. Ligands partial charges were determined by using the AM1-BCC method available in the AMBERTOOLS package. The systems were solvated in explicit water using the TIP3P model in a box 20 Å, then, saline solution with physiological concentration (0.15 M) was added, ensuring system neutrality. NAMD software version 2.13 (8) was used to perform simulations. Periodic boundary conditions were used with a 12 Å for non-bonded interactions. Long-range electrostatic corrections were considered by particle mesh Ewald summation. In addition, restriction of vibration of covalent bonds involving hydrogen atoms, HOH angles, and the OH bond distance of TIP3P water molecules was used, and a time-step equal to 2 fs. Initially, a minimization scheme was applied to the system, which was then gradually heated up to 310 K by applying to decrease harmonic restraining forces in three stages: (i) the system was heated up to 100K in 40-ps considering harmonic restraints with a force constant of 30 kcal/mol·Å^2 on cα atoms and 10 kcal/mol·Å^2 on C and N atoms of the protein backbone; (ii) the system was simulated for 100-ps with a force constant of 15 kcal/mol·Å^2 on Cα atoms and 5 kcal/mol·Å2 on C and N atoms of the protein backbone for 100-ps; (iii) the temperature

of the system was increased to the desired value of 310K after simulating for 200-ps by keeping weak harmonic restraints with a force constant of 7,5 kcal/mol·Å² on Cα atoms and of 2.5 kcal/mol·Å² on C and N atoms of the protein backbone. The thermalization phase (NVT ensemble) was followed by a pressurization phase (NPT ensemble). The system was then simulated for 100-ps, there were weak harmonic restraints with a force constant of 3,75 kcal/mol·Å² on Cα atoms and 1,25 kcal/mol·Å² on C and N atoms. Then a stage of 200-ps was carried out and only Cα atoms of the protein were restrained with a force constant of ~1,5 kcal/mol·Å². A pressure of 1 atm was maintained using the Langevin piston barostat and the system was simulated for 100-ns without restraints.

Metadynamics Simulations. The final structures from the molecular dynamics simulations were used as a starting point for metadynamics calculations using Plumed version 2.0.1 (9) within NAMD version 2.13 (8). Unbinding was driven by a collective variable (CV). The CV was defined as the distance between the center of mass of the CZV1 compounds and mAbs. A potential bias in the form of a Gaussian with width and height equal to 0.05 nm and 0.05 kJ/mol, respectively, was added along the y-axis projection in 1 ps increments until complete unbinding. Positional constraints were used on the Cα atoms of the mAbs using a force constant of 1 kcal/mol to avoid artificial distortions of the protein structure during the uncoupling process. The uncoupling for all simulations occurred during 10 ns of simulation.

Computational alanine scanning calculations. The computational alanine scanning protocol available in Rosetta v.3.12 (10) was used to identify hotspot residues in the protein-protein and protein-ligand complexes. The Rosetta software was also used to predict the binding affinity (ddG) of these complexes. All protocols were run through the RosettaScripts application, which in turn uses an XML file as input. All calculations were performed using the beta_nov16 potentials and the geometry from the last frame of the molecular dynamics simulation. The ligand parameters were generated using the molfile_to_params.py script available in the Rosetta script suite.

Data, Materials, and Software Availability.
All study data are included in the article and/or in the SI Appendix. Sequencing data generated in this study are deposited in the Gene Expression Omnibus (GEO) dataset under the accession number GSE254062.

Supplementary Figures

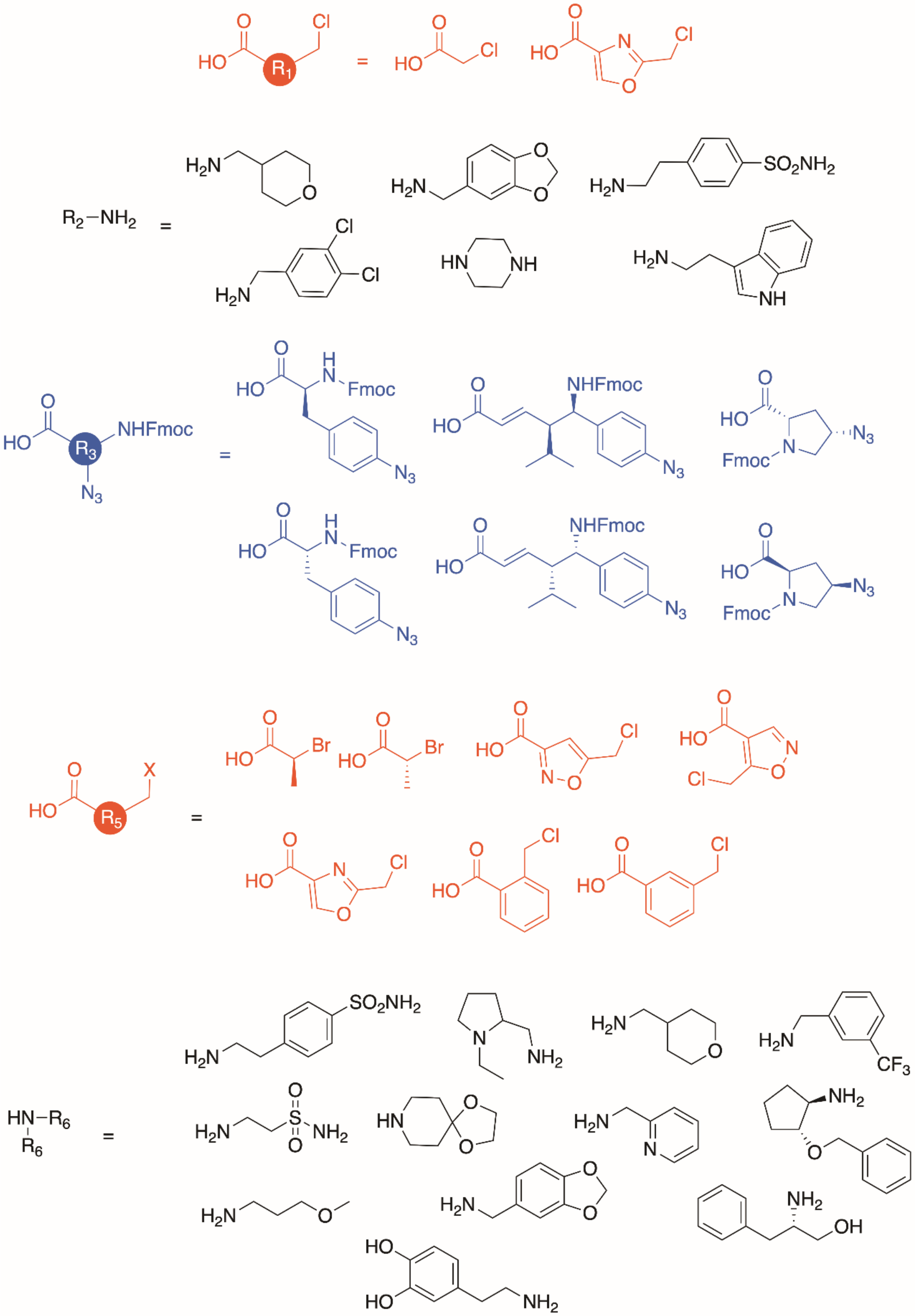

Fig. S1. Chemical structures of the building blocks used at the R1, R2, R3, R5, and R6 positions (related to Figure 1).

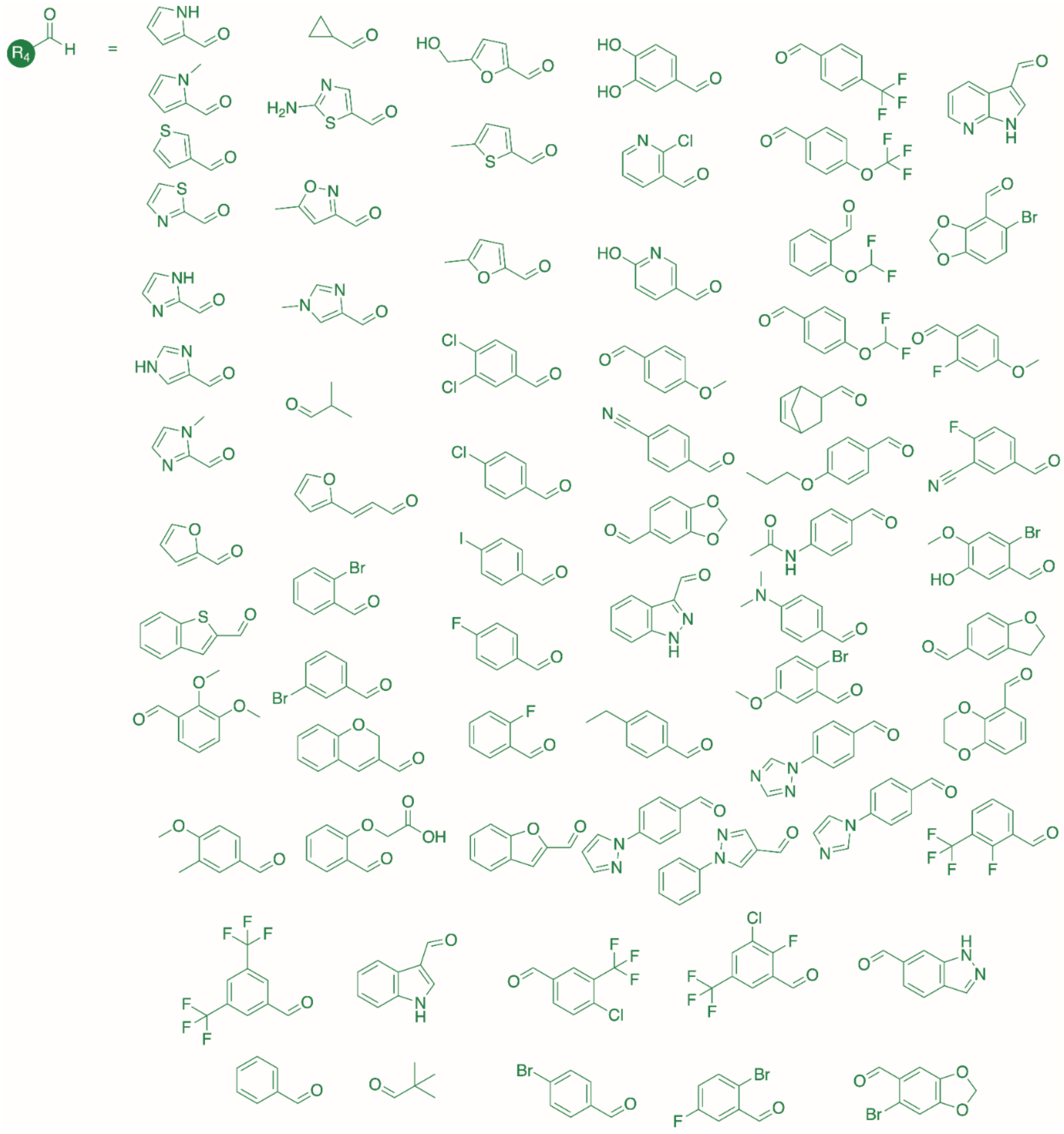

Fig. S2. Chemical structures of the aldehydes used to install the R4 unit (related to Figure 1).

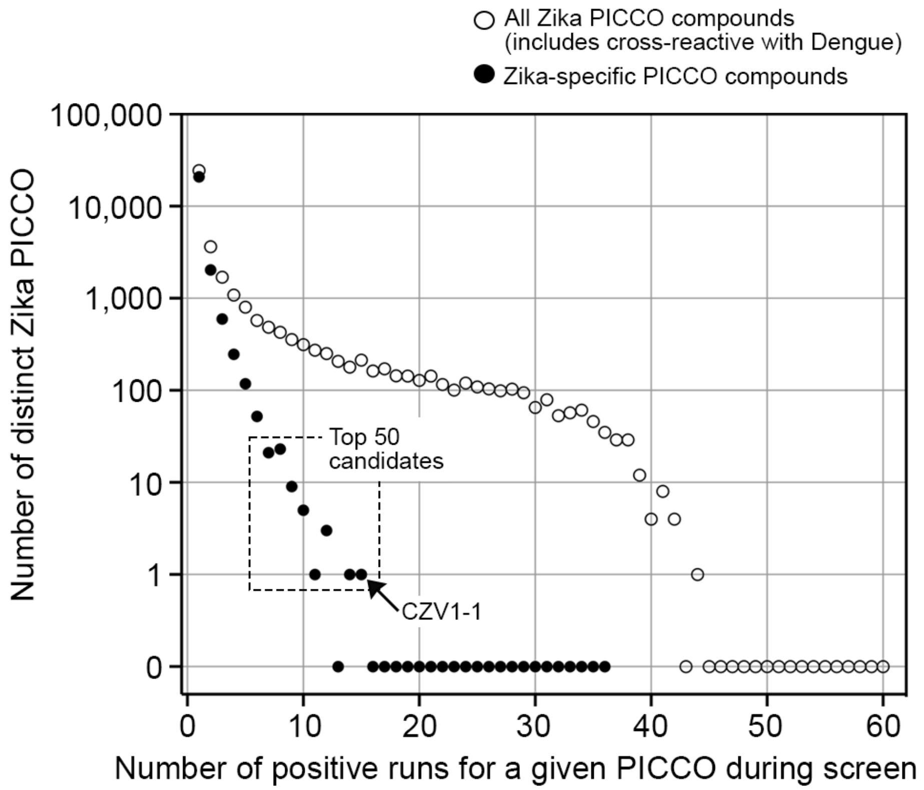

Fig. S3. Number of distinct PICCO compounds detected once, twice, or multiple times across Zika discovery runs.

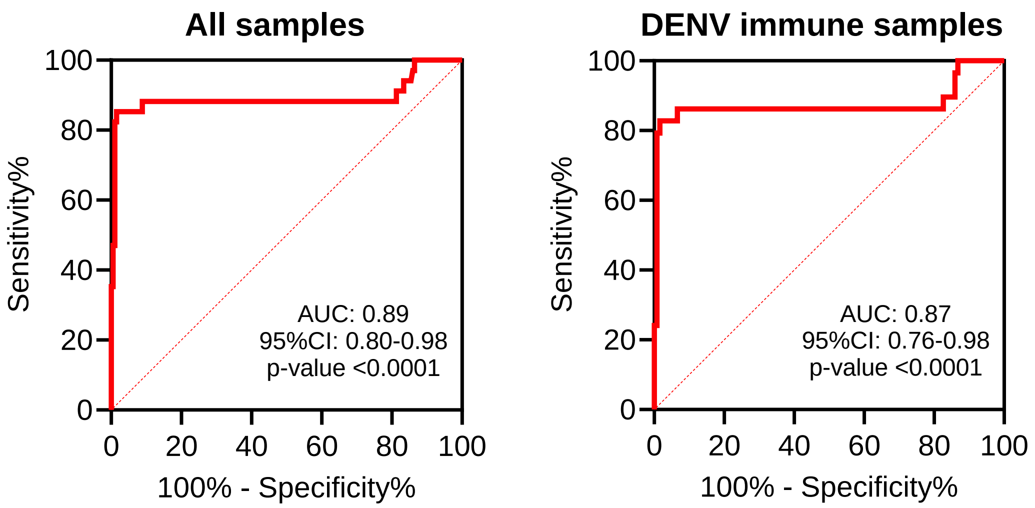

Fig. S4. Validation of CZV1-1 as a Zika diagnostic marker. For this analysis we have used a larger set of serum samples (n=266) with a broad repertoire of flavivirus immune reactivity. The receiver operating characteristics (ROC) curve for all samples and the DENV-immune patients is shown. The calculated area under the curve (AUC) and 95% confidence intervals (95% CI) are depicted. DENV, dengue virus.

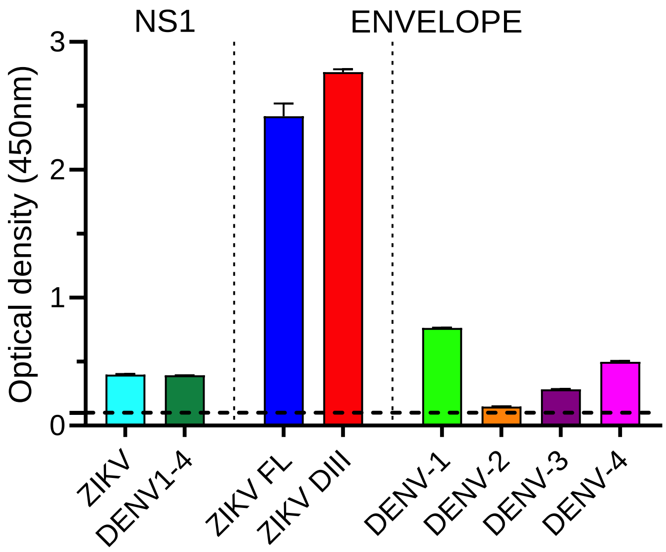

Fig. S5. Binding of purified IgG against CZV1-1 against ZIKV and DENV structural and non-structural proteins. The dotted line along y-axis represents blank average for the binding assay. DENV, dengue virus; ZIKV, Zika virus; ZIKV FL, Full-length envelope protein; ZIKV DIII, domain III of ZIKV envelope protein.

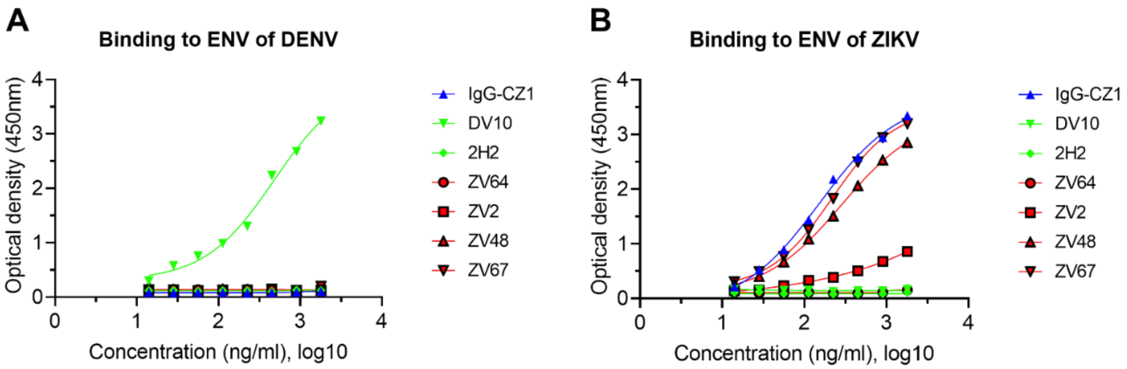

Fig. S6. Binding of purified IgG against CZV1-1 and several Zika-specific (ZV2, ZV48, ZV64, ZV67) or dengue-specific (DV10 and 2H2) monoclonal antibodies against dengue virus (A) and ZIKV (B) envelope proteins. ENV, envelope protein; ZIKV, Zika virus.

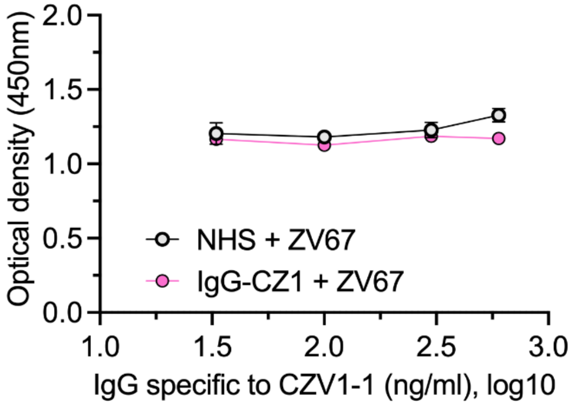

Fig. S7. Reverse blocking experiments to assess the competition between the ZV67 mAb and IgG specific to CZV1-1 for binding to the ZIKV envelope. NHS, normal human serum.

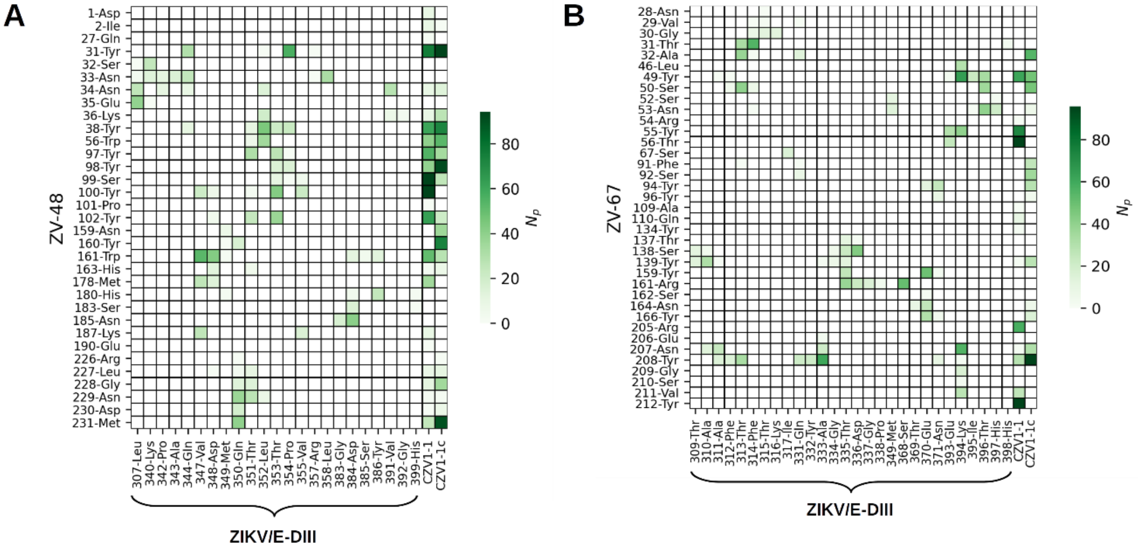

Fig. S8. Heatmap showing the native contact's profile between mAbs and ligands. Np comprises the total number of atom pairs that make contact along the molecular dynamics simulations. **(A)** native contact profile for ZV48 and ZIKV E-DIII. E-DIII makes contact with the following residues of mAb ZV48: Y31, S32, N33, N34, E35, K36, Y38, W56, Y97, Y98, S99, Y100, P101, Y102, N159, Y160, W161, H163, M178, H180, S183, N185, K187, E190, R226, L227, G228, N229, D230, and M231; whereas CZV1-1 adduct makes contact with Y31, S32, N33, N34, E35, K36, Y38, W56, Y97, Y98, S99, Y100, P101, Y102, N159, Y160, W161, H163, M178, H180, S183, N185, K187, E190, R226, L227, G228, N229, D230, and M231. The CZV1-1c adduct contacts Y31, N34, K35, Y38, W56, Y97, Y98, S99, Y102, N159, Y160, W161, G228, and M231. **(B)** native contact profile for ZV67 and ZIKV E-DIII. E-DIII makes contact with the following residues of mAb ZV67: N28, V29, G30, T31, A32, L46, Y49, S50, N53, R54, Y55, T56, S67, F91, S92, Y94, Y96, A109, Q110, Y134, T137, S138, Y139, Y159, R161, S162, N164, Y166, R205, E206, N207, Y208, G209, S210, V211, Y212; while CZV1-1 makes contact with Y49, Y55, T56, R205, Y208, V211 and Y212. The CZV1-1c adduct contacts A32, Y49, S50, F91, S92, Y94, Y139, N207 and Y208). ZIKV, Zika virus; E-DIII, domain III of ZIKV envelope protein.

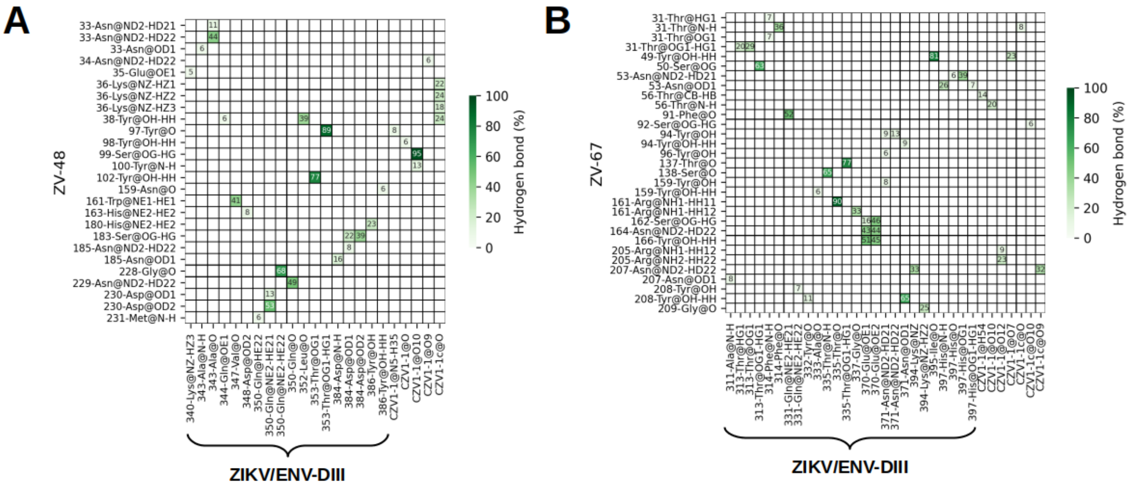

Fig. S9. Heatmap showing the hydrogen bonds profile. **(A)** Hydrogen bond profile between ZV48/DIII and ZV48/CZV1-1 adducts 1 and 1c; **(B)** hydrogen bond profile between ZV67/DIII and ZV67/CZV1 adducts 1 and 1c. ZIKV, Zika virus; E-DIII, domain III of ZIKV envelope protein.

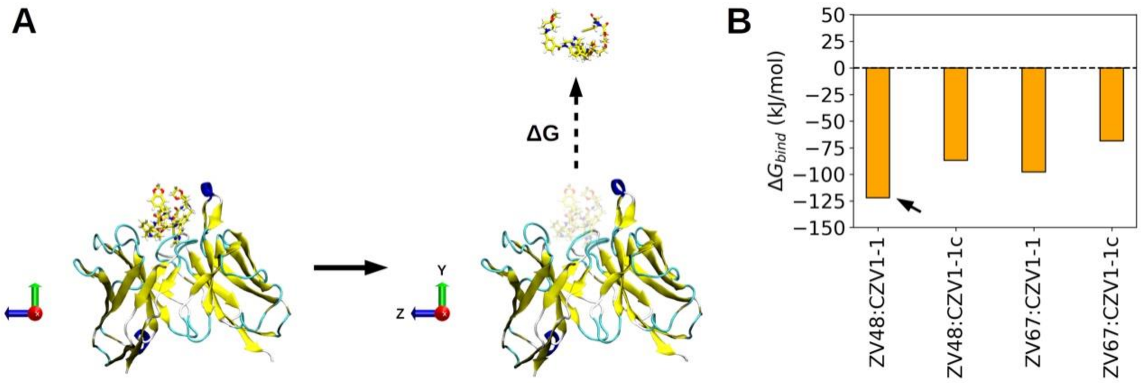

Fig. S10. Binding free energy profile of mAb/CZV1 complexes. (A) Illustration showing the dissociation pathway of CZV1-1 adducts. On the left, the adduct is bound to mAb, while on the right (at the end of the simulation) it is fully uncoupled. (B) Bar graph showing the binding free energy minima for the mAb/CZV1-1 complexes. The black arrow indicates the compound that obtained the most negative binding free energy, and thus the strongest association.

Supplementary Tables

Table S1. Serological characterization of the Zika immune samples. Samples were used to generate ten pools of two patients each and used at the screening step of the PICCOs library.

Patient ID	Year of sample collection	Age	Gender	Binding assays (ELISA)			Neutralization assays, antibody titer				
				ZIKV IgM	Dengue IgM	Dengue IgG	ZIKV	DENV-1	DENV-2	DENV-3	DENV-4
01-007-2-2	2016	19	Female	NEG	NEG	NEG	140.0	<20	<20	<20	<20
01-008-2-2	2016	25	Female	NEG	NEG	NEG	276.6	<20	<20	<20	<20
01-009-2-2	2016	27	Female	NEG	NEG	NEG	529.1	<20	<20	<20	<20
03-006-2-2	2016	28	Female	NEG	NEG	NEG	672.6	<20	<20	<20	<20
62-0005	2015	36	Female	POS	NEG	NEG	980.4	<20	<20	<20	<20

NEG, negative; POS, positive.

Table S2. Serological characterization of the dengue immune samples included in the initial screening step to identify and exclude ligands that bind to cross-reactive, flavivirus IgG antibodies. All patients were classified as dengue primary cases. Matched acute and convalescent sera were pooled to form five matched pools of 2-4 patients.

Patient ID	Days from start of symptoms	Year of sample collection	Age	Sex	IgM DENV ENV	IgG DENV ENV	IgG NS1	Group category
287	3	2005	16	F	NEG	NEG	49	Dengue 1, Acute
287	5	2005	16	F	NEG	NEG	49	
287	31	2005	16	F	POS	POS	17074	Dengue 1, convalescent
287	324	2006	16	F		POS	4479	
263	2	2005	25	F	NEG	NEG	49	Dengue 1, Acute
263	5	2005	25	F	NEG	NEG	49	
263	21	2005	25	F	POS	POS	2235	Dengue 1, convalescent
263	324	2006	25	F		POS	5090	
295	5	2005	18	M	POS	NEG	49	Dengue 2, Acute
295	26	2005	18	M	POS	POS	245013	Dengue 2, convalescent
295	34	2005	18	M	POS	POS	3220	
295	46	2005	18	M	POS	POS	2749	
249	5	2005	43	M	NEG	NEG	49	Dengue 2, Acute
249	7	2005	43	M	POS	NEG	86	
249	9	2005	43	M	POS	NEG	98	Dengue 2, convalescent
249	33	2005	43	M	POS	POS	20264	
299	7	2005	56	M	POS	NEG	49	Dengue 2, Acute
299	16	2005	56	M	POS	POS	4706	Dengue 2, convalescent
299	297	2006	56	M	POS	POS	2169	
574	4	2006	30	F	POS	NEG	88	Dengue 3, Acute
574	27	2006	30	F	POS	POS	18558	Dengue 3, convalescent
574	48	2006	30	F	POS	POS	24840	
576	5	2006	66	F	NEG	NEG	89	Dengue 3, Acute
576	33	2006	66	F	POS	POS	5368	Dengue 3, convalescent
576	383	2007	66	F		POS	2670	
279	3	2005	17	F	NEG	NEG	49	Dengue 3, Acute
279	36	2005	17	F	POS	POS		Dengue 3, convalescent
279	304	2006	17	F		POS		
396	2	2006	10	M	NEG	NEG	49	Dengue 4, Acute
396	7	2006	10	M	NEG	NEG	64.5	
396	29	2006	10	M	POS	POS	14235	Dengue 4, convalescent
577	4	2006	8	F	POS	NEG	49	Dengue 4, Acute

577	29	2006	8	F	POS	POS	28350	Dengue 4, convalescent
393	5	2006	6	F	POS	NEG	49	Dengue 4, Acute
393	26	2006	6	F	POS	POS	5942	Dengue4, convalescent
394	8	2006	8	M	POS	NEG	49	Dengue 4, Acute
394	39	2006	8	M	NEG	POS	1859	Dengue 4, convalescent
327	2	2005	35	M	NEG	NEG	49	Dengue 5, Acute
327	4	2005	35	M	NEG	NEG	49	
327	31	2005	35	M	POS	POS	1216	Dengue 5, convalescent
527	2	2006	38	M	NEG	NEG	49	Dengue 5, Acute
527	6	2006	38	M	NEG	NEG	62.3	
527	31	2006	38	M	POS	POS	4919	Dengue 5, convalescent
658	4	2006	27	F	NEG	NEG	49	Dengue 5, Acute
658	6	2006	27	F	POS	NEG	49	
658	33	2006	27	F	POS	POS	6296	Dengue 5, convalescent
658	437	2007	27	F		POS	674	
644	4	2006	48	F	NEG	NEG	49	Dengue 5, Acute
644	6	2006	48	F	POS	NEG	49	
644	33	2006	48	F	POS	POS	1423	Dengue 5, convalescent
644	403	2007	48	F		POS	1505	

DENV ENV, dengue virus envelope; F, female; M, male; POS, positive; NEG, negative

Table S3. Serological characterization of the Zika and dengue naïve samples included in the screening step of the PICCOs library. Three pools of three samples each were included in this step.

Group	Patient ID	Year of sample collection	Age	Sex	Binding assays (ELISA)		Neutralization assays, antibody titer			
					Dengue IgM	Dengue IgG	DENV-1	DENV-2	DENV-3	DENV-4
Naïve 1	B021	2011	24	Female	NEG	NEG	<20	<20	<20	<20
	B036	2011	16	Female	NEG	NEG	<20	<20	<20	<20
	B235	2011	16	Female	NEG	NEG	<20	<20	<20	<20
Naïve 2	B266	2011	27	Female	NEG	NEG	<20	<20	<20	<20
	B282	2011	14	Female	NEG	NEG	<20	<20	<20	<20
	B313	2012	16	Female	NEG	NEG	<20	<20	<20	<20
Naïve 3	B357	2012	18	Female	NEG	NEG	<20	<20	<20	<20
	B084	2011	21	Female	NEG	NEG	<20	<20	<20	<20
	B215	2011	21	Female	NEG	NEG	<20	<20	<20	<20

NEG, negative.

Table S4. Summary of PICCOs selected during the screening step.

	Beads Screened by FACS	Beads Positive by FACS	Total Unique PICCOs detected	Total Unique Specific PICCOs detected*	High Hit Specific PICCOs Selected for Clustering**	PICCOs Specific Clusters Identified	Unique PICCO selected for Detailed Study
Zika	150 million (60 runs)	171,309	37,291	3,102	50	5	CZV1-1
Dengue	75 million (30 runs)	128,942	30,789	2,516	50	3	-
Flavivirus	-	-	-	-	50	8	-

* Zika Specific: Positive ≥ 2 runs, zero dengue runs; Dengue Specific: Positive ≥ 2 runs, zero Zika runs

****** Zika Specific: Positive ≥ 7 runs, zero dengue runs; Dengue Specific: Positive ≥ 5 runs, zero Zika runs; Flavivirus cross-reactive: Positive ≥ 26 dengue runs and ≥ 38 Zika runs

Table S5. Summary of the number of PICCOs identified following hierarchical clustering.

Clusters Zika Specific	Number of Unique PICCOs in Cluster	Number of Runs in which Each Unique PICCO was Detected	Mean (STDV) of Number Runs Detected
CZV1	11	15,14,11,10,9,8,8,8,8,8,7	9.6 (2.7)
CZV2	8	12,10,10,9,9,8,7,7	9.2 (1.5)
CZV3	8	12,10,9,9,9,8,8,7	9.0 (1.5)
CZV4	10	10,8,8,8,8,8,8,7,7,7	7.9 (0.9)
CZV5	3	8,8,8	8
No Cluster	10	12,9,9,8,8,8,8,8,7,7	8.4 (1.4)

Table S6. Serological characterization of serum samples for the validation step.

Groups	Patient ID	Year of sample collection	Age	Binding assays (ELISA)			Neutralization assays, antibody titer				
				Dengue IgM	Dengue IgG	ZIKV IgM	DENV-1	DENV-2	DENV-3	DENV-4	ZIKV
Zika 1	62-0020	14		NEG	NEG	POS	<20	<20	<20	<20	1227
	1	-		NEG	NEG	NEG	<20	<20	<20	<20	2560
	5	-		NEG	NEG	NEG	<20	<20	<20	<20	12980
Zika 2	62-0035	16		NEG	NEG	POS	<20	<20	<20	<20	11569
	13	-		NEG	NEG	EQUIV	<20	<20	<20	<20	1575
	1	-		NEG	NEG	NEG	<20	<20	<20	<20	2560
	41	-		NEG	NEG	EQUIV	<20	<20	<20	<20	343
Dengue 1	514 S4	2010	12	POS	POS	NEG					<20
	273 S2	2010	58	POS	POS	NEG					<20
	426 S3	2010	15	POS	POS	NEG					<20
Dengue 2	270 S2	2010	27	POS	POS	NEG					<20
	304 S3	2010	71	POS	POS	NEG					<20
	139 S4	2010	75	POS	POS	NEG					<20
Dengue 3	235 S3	2010	70	POS	POS	NEG					<20
	197 S3	2010	25	POS	POS	NEG					<20
	502 S4	2010	15	POS	POS	NEG					<20
Dengue 4	515 S3	2010	80	POS	POS	NEG					<20
	257 S2	2010	8	POS	POS	NEG					<20
	183 S4	2010	26	POS	POS	NEG					<20
Flavivirus 1	62-0071	18		NEG	POS	POS	<20	92	1004	3251	4174
	1	-		NEG	NEG	NEG	<20	<20	<20	<20	2560
	7	-		NEG	POS	POS	<20	<20	80	<20	2560
Flavivirus 2	23	-		NEG	NEG	NEG	<20	<20	<20	<20	573
	41	-		NEG	NEG	EQUIV	<20	<20	<20	<20	43
	62-0028	11		POS	POS	POS	<20	<20	1282	1387	14092

NEG, negative; POS, positive; EQUIV, equivocal.

Table S7. Docking scores for the adducts CZV1-1 and CZV1-1c bound to different ZIKV-neutralizing mAbs ZV48, ZV67 and Z004.

Neutralizing mAb	Ligand	GOLD score
ZV48	E-DIII	N/A*
	CZV1-1	74.10
	CZV1-1c	61.23
ZV67	E-DIII	N/A*
	CZV1-1	65.19
	CZV1-1c	60.63
Z004	CZV1-1	47.57
	CZV1-1c	46.08

*Gold software is not suitable for protein-protein docking.
E-DIII, domain III of Zika virus envelope protein.

References

1. P. M. S. Castanha, *et al.*, Placental Transfer of Dengue Virus (DENV)–Specific Antibodies and Kinetics of DENV Infection–Enhancing Activity in Brazilian Infants. *Journal of Infectious Diseases* **214**, 265–272 (2016).
2. G. Jones, P. Willett, R. C. Glen, A. R. Leach, R. Taylor, Development and validation of a genetic algorithm for flexible docking 1 1Edited by F. E. Cohen. *J Mol Biol* **267**, 727–748 (1997).
3. H. Zhao, *et al.*, Structural Basis of Zika Virus-Specific Antibody Protection. *Cell* **166**, 1016–1027 (2016).
4. D. F. Robbiani, *et al.*, Recurrent Potent Human Neutralizing Antibodies to Zika Virus in Brazil and Mexico. *Cell* **169**, 597-609.e11 (2017).
5. J. A. Maier, *et al.*, ff14SB: Improving the Accuracy of Protein Side Chain and Backbone Parameters from ff99SB. *J Chem Theory Comput* **11**, 3696–3713 (2015).
6. J. Wang, R. M. Wolf, J. W. Caldwell, P. A. Kollman, D. A. Case, Development and testing of a general amber force field. *J Comput Chem* **25**, 1157–1174 (2004).
7. T. J. Dolinsky, J. E. Nielsen, J. A. McCammon, N. A. Baker, PDB2PQR: an automated pipeline for the setup of Poisson-Boltzmann electrostatics calculations. *Nucleic Acids Res* **32**, W665–W667 (2004).
8. J. C. Phillips, *et al.*, Scalable molecular dynamics with NAMD. *J Comput Chem* **26**, 1781–1802 (2005).
9. The PLUMED consortium, Promoting transparency and reproducibility in enhanced molecular simulations. *Nat Methods* **16**, 670–673 (2019).
10. T. Kortemme, D. E. Kim, D. Baker, Computational Alanine Scanning of Protein-Protein Interfaces. *Science's STKE* **2004** (2004).
